# Anthranilate Fluorescence Marks a Calcium-Propagated Necrotic Wave That Promotes Organismal Death in *C. elegans*

**DOI:** 10.1371/journal.pbio.1001613

**Published:** 2013-07-23

**Authors:** Cassandra Coburn, Erik Allman, Parag Mahanti, Alexandre Benedetto, Filipe Cabreiro, Zachary Pincus, Filip Matthijssens, Caroline Araiz, Abraham Mandel, Manolis Vlachos, Sally-Anne Edwards, Grahame Fischer, Alexander Davidson, Rosina E. Pryor, Ailsa Stevens, Frank J. Slack, Nektarios Tavernarakis, Bart P. Braeckman, Frank C. Schroeder, Keith Nehrke, David Gems

**Affiliations:** 1 Institute of Healthy Ageing, and Research Department of Genetics, Evolution and Environment, University College London, London, United Kingdom; 2 Department of Pharmacology and Physiology, University of Rochester Medical Center, Rochester, New York, United States of America; 3 Boyce Thompson Institute and Department of Chemistry and Chemical Biology, Cornell University, Ithaca, New York, United States of America; 4 Department of Molecular, Cellular and Developmental Biology, Yale University, New Haven, Connecticut, United States of America; 5 Department of Biology, Ghent University, Ghent, Belgium; 6 Institute of Molecular Biology and Biotechnology, University of Crete, Heraklion, Crete, Greece; 7 Department of Medicine, University of Rochester Medical Center, Rochester, New York, United States of America; St. Jude Children's Research Hospital, United States of America

## Abstract

For cells the passage from life to death can involve a regulated, programmed transition. In contrast to cell death, the mechanisms of systemic collapse underlying organismal death remain poorly understood. Here we present evidence of a cascade of cell death involving the calpain-cathepsin necrosis pathway that can drive organismal death in *Caenorhabditis elegans*. We report that organismal death is accompanied by a burst of intense blue fluorescence, generated within intestinal cells by the necrotic cell death pathway. Such death fluorescence marks an anterior to posterior wave of intestinal cell death that is accompanied by cytosolic acidosis. This wave is propagated via the innexin INX-16, likely by calcium influx. Notably, inhibition of systemic necrosis can delay stress-induced death. We also identify the source of the blue fluorescence, initially present in intestinal lysosome-related organelles (gut granules), as anthranilic acid glucosyl esters—not, as previously surmised, the damage product lipofuscin. Anthranilic acid is derived from tryptophan by action of the kynurenine pathway. These findings reveal a central mechanism of organismal death in *C. elegans* that is related to necrotic propagation in mammals—e.g., in excitotoxicity and ischemia-induced neurodegeneration. Endogenous anthranilate fluorescence renders visible the spatio-temporal dynamics of *C. elegans* organismal death.

## Abstract 2

Death of the nematode *Caenorhabditis elegans* involves a conserved necrotic cell death cascade which generates endogenous blue anthranilate fluorescence, allowing death to be visualized.

## Author Summary

In the nematode *Caenorhabditis elegans*, intestinal lysosome-related organelles (or “gut granules”) contain a bright blue fluorescent substance of unknown identity. This has similar spectral properties to lipofuscin, a product of oxidative damage known to accumulate with age in postmitotic mammalian cells. Blue fluorescence seems to increase in aging worm populations, and lipofuscin has been proposed to be the source. To analyze this further, we measure fluorescence levels after exposure to oxidative stress and during aging in individually tracked worms. Surprisingly, neither of these conditions increases fluorescence levels; instead blue fluorescence increases in a striking and rapid burst at death. Such *death fluorescence* (DF) also appears in young worms when killed, irrespective of age or cause of death. We chemically identify DF as anthranilic acid glucosyl esters derived from tryptophan, and not lipofuscin. In addition, we show that DF generation in the intestine is dependent upon the necrotic cell death cascade, previously characterized as a driver of neurodegeneration. We find that necrosis spreads in a rapid wave along the intestine by calcium influx via innexin ion channels, accompanied by cytosolic acidosis. Inhibition of necrosis pathway components can delay stress-induced death, supporting its role as a driver of organismal death. This necrotic cascade provides a model system to study neurodegeneration and organismal death.

## Introduction

While mechanisms of cell death such as apoptosis are well characterized [1], less is known about the mechanisms of organismal death, particularly in invertebrate model organisms. Here we investigate organismal death in the nematode *C. elegans*, using a newly discovered, endogenous fluorescent marker of death.

One possibility is that organismal death results from a cascade of cell death. As first defined by Kerr et al. in 1972 [1], cell death has been viewed as taking two forms: controlled (apoptotic) or uncontrolled (necrotic). However, more recent elucidation of the mechanisms underlying necrotic cell death reveals that it too can be a regulated process [2]–[5]. Biochemical hallmarks of necrosis include calcium-mediated initiation, lysosomal membrane permeabilization (LMP), and activation of noncaspase proteases (calpains and cathepsins) [5]–[7].

Necrosis as a regulated process has been characterized mainly in mammalian neuronal models. Excitotoxic neuronal cell death occurs in response to overstimulation with the excitatory neurotransmitter glutamate (e.g., under conditions of ischemia or stroke) [7]. Sustained activation of glutamate receptors causes a cytosolic influx of extracellular Ca^2+^ [8]. Increased Ca^2+^ levels lead to cell death, largely through activation of associated proteases [9]. Moreover, Ca^2+^ may spread between cells via connecting gap junctions, and gap junction inhibition reduces ischemia-induced neurodegeneration [10],[11].

Through the study of ischemia-induced death in mammalian CA1 hippocampal neurons, Yamashima and co-workers identified the calpain-cathepsin cascade as an effector of necrotic cell death. Ischemia increases intracellular Ca^2+^ levels, which activate Ca^2+^-dependent cysteine proteases (calpains) [12]. These calpains cause lysosomal lysis, leading to cytosolic acidosis and the destructive release of lysosomal cathepsin proteases [13].

Many components of the calpain-cathepsin cascade are present in *C. elegans*, where necrotic cell death can be induced in neurons by mutations such as *mec-4(u231)* [14]. For example, *mec-4*-induced neurodegeneration requires the calcium-dependent calpains TRA-3 and CLP-1 and the cathepsins ASP-3 and ASP-4 [15].

LMP is a central event in the necrotic cascade, and the degree of LMP can influence the cellular decision to live or to die via necrosis or apoptosis [3],[5],[16]. In *C. elegans*, lysosomes are required for osmotic stress-induced necrotic death [17] and interventions that increase lysosomal pH can ameliorate *mec-4(d)*-induced neurodegeneration [18].

**Figure 1:**
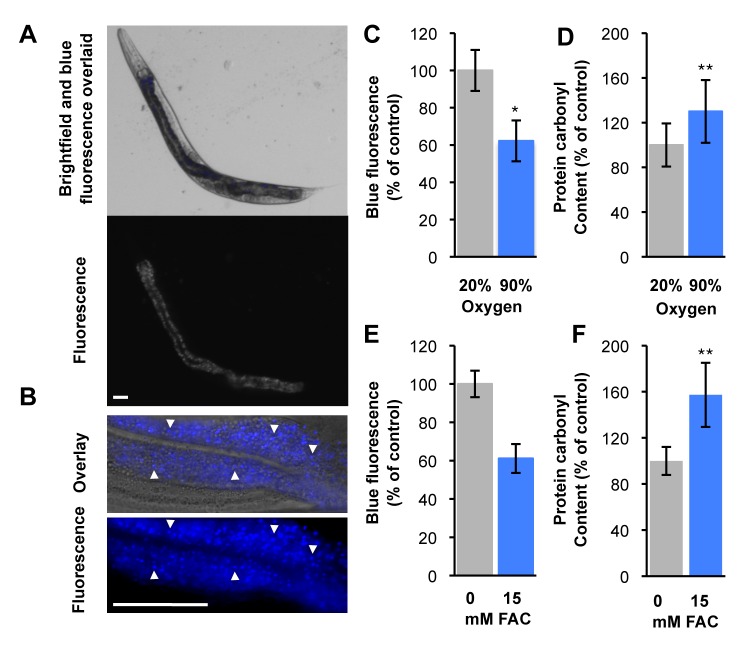
Molecular damage does not increase blue fluorescence. (A, B) Fluorescent gut granules (arrow heads) in intestinal cells of healthy, young adult *C. elegans*. Bar, 50 µm. (C–F) Hyperoxia (5-d exposure) and free iron (1-d exposure) increase protein oxidation but not blue fluorescence, mean of 3 biological replicates ± SEM, * *p*<0.05, ** *p*<0.01.

*C. elegans* intestinal cells contain both lysosomes and gut granules, which are large, melanosome-like lysosome-related organelles [19]. Under ultraviolet light, gut granules emit blue fluorescence, with maximal intensity at λ_ex_/λ_em_ 340/430 nm (Figure 1A–B) [20]. This fluorescence has been attributed to lipofuscin [21],[22], a heterogeneous, cross-linked aggregate of oxidatively damaged lipids and proteins. Lipofuscin accumulates with age in postmitotic mammalian cells and so has frequently been used as a biomarker of aging [23]–[25]. Lipofuscin composition is highly variable but can be identified by virtue of its autofluorescence [24]. If excited by UV light *in vitro* it emits blue fluorescence, which may reflect formation of fluorescent Schiff bases between carbonyl and amino groups [26],[27]. However, UV excitation of lipofuscin *in vivo* results in peak fluorescence in the 540–640 nm (orange-yellow) range [28].

Several observations have led to the suggestion that the fluorescent material in the *C. elegans* intestine is lipofuscin. Its fluorescence peak at λ_ex_/λ_em_ 340/430 nm is similar to that of lipofuscin *in vitro*, it is localized to the lysosome-like gut granules, and its levels increase in aging populations [20]–[22],[29]. It is often used as a biomarker of aging—for example, to verify that treatments that shorten worm lifespan do so by accelerating aging. The presence of lipofuscin in *C. elegans* would support the view that aging is caused by accumulation of molecular damage. Yet it remains possible that the fluorescent substance in gut granules is not lipofuscin. For example, studies of *flu* mutations causing altered gut granule fluorescence suggest that it corresponds to fluorescent tryptophan metabolites [30].

In this study, we describe how a reassessment of blue fluorescence in *C. elegans* led to the discovery of the phenomenon of death fluorescence (DF), a burst of blue fluorescence that accompanies death in *C. elegans*. We establish that both DF and gut granule fluorescence originate not from lipofuscin, but from tryptophan-derived anthranilic acid glucosyl esters. We then show that DF is generated by the calpain-cathepsin necrotic cell death pathway, and requires calcium signaling for organismal propagation. Finally, we show that inhibition of this pathway can protect animals against stress-induced death, supporting a role of systemic necrotic cell death in organismal death.

## Results

### Oxidative and Thermal Stress Do Not Increase Blue Fluorescence

Lipofuscin is formed through accumulation of oxidatively damaged proteins and lipids [24]. For example, raised oxygen level (40% O_2_) increases lipofuscin levels in human fibroblasts [25]. To probe whether the blue fluorescent material in *C. elegans* gut granules (Figure 1A–B) is lipofuscin, we exposed them to normobaric hyperoxia (90% O_2_), and elevated iron levels. Both treatments significantly increased protein oxidative damage but neither increased blue fluorescence levels (Figure 1C–F). Elevated expression of *hsp-4::gfp* is indicative of the unfolded protein response [31], symptomatic of protein damage. Heat shock increased *hsp-4::gfp* expression but not blue fluorescence (Figure S1). These results imply that *C. elegans* blue fluorescence is not generated by oxidative damage, suggesting that it is not lipofuscin.

### A Burst of Blue Fluorescence Occurs When *C. elegans* Die

Like lipofuscin in mammals, mean fluorescence levels rise gradually with age in *C. elegans* population cohorts [20],[29]. However, population mean data do not address heterogeneity in the fluorescence of individual worms. This concern was raised by a previous study [20], as follows. Aging worms can be classed according to their degree of motility: class A animals move normally, class B animals move more slowly, and class C animals do not move away when touched, and are near to death [32]. Notably, blue fluorescence levels did not differ significantly between class A and B, and only increased in class C worms [20]. This suggests that blue fluorescence levels in worms increase only as they approach death.

**Figure 2:**
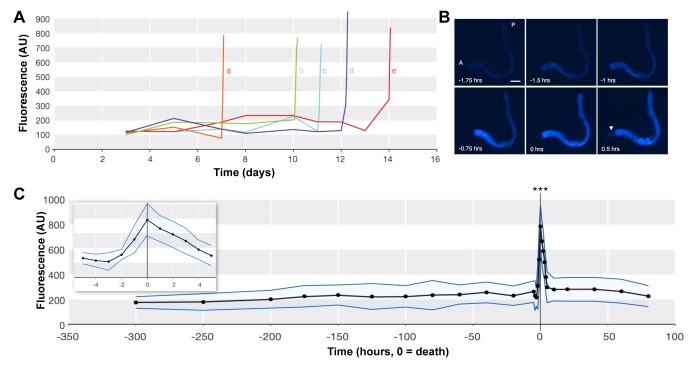
Blue fluorescence increases with death. (A) Fluorescence levels in individual animals (a–e) during life and death. (B) Typical fluorescence change during death from old age. 0 h, cessation of movement (death). All six images are of the same dying animal. Scale bar, 50 µm. (C) Mean levels of fluorescence relative to time of death (black line), ± SD (blue lines). Data from 47 individuals. Inset, death peak detail. AU, arbitrary units.

To test this directly, fluorescence levels of individually cultured, wild-type *C. elegans in situ* on nematode growth medium (NGM) agar plates were examined at intervals throughout life (DAPI filter; λ_ex_/λ_em_ 350/460 nm). As animals approached death (as indicated by reduced movement), time-lapse imaging was used to capture fluorescence changes during death. This revealed that fluorescence levels in individual animals change little until immediately prior to death. A striking and sudden ∼400% increase in fluorescence level then occurs, coinciding with cessation of movement (i.e., death) (Figure 2; Video S1). This rise begins at ∼2 h prior to death, and then fades by ∼6 h after death (Figure 2B–C).

**Figure 3:**
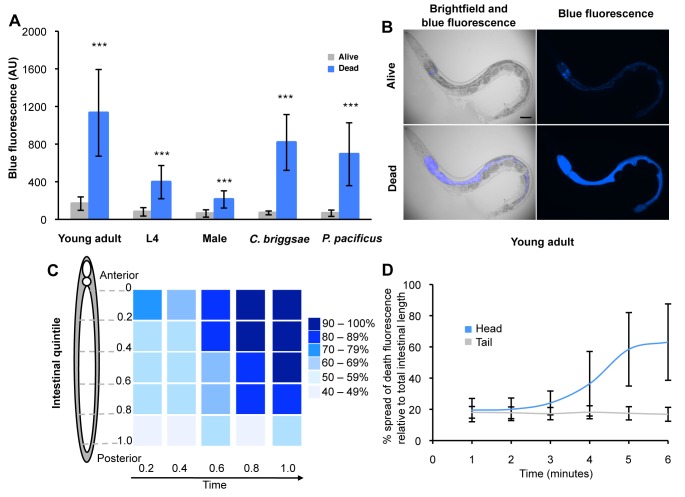
Characteristics of DF. (A) DF induced by hot pick killing in young WT adult (*N* = 87), L4 (*N* = 26), and male *C. elegans* (*N* = 25), and in other nematode species *C. briggsae* (*N* = 26) and *P. pacificus* (*N* = 49). ± SD, ***p<0.001. L4 larvae and adult males show smaller increases in DF than adult hermaphrodites, perhaps due to their smaller size. (B) Typical fluorescence increase in young adult worm killed by a hot pick. (C) Fluorescence increases in an anterior to posterior wave. Figure shows mean fluorescence intensity (values normalized to highest value in each individual) at 5 points along the intestine of animals dying of old age (*N* = 29). Due to individual variation (range, 1 h 55 min–5 h 30 min, mean ∼3 h), the duration of each observation was divided into 5 equal points for measurement. T = 0 is the point at which time-lapse photography of dying (late stage C) worms was initiated. (D) DF will not propagate in a posterior to anterior wave. Head, int1 and int2 anterior intestinal cells; tail, int9 posterior intestinal cells.

Blue fluorescent bursts are not only associated with death from old age, but were also induced by killing—for example, by placing a heated worm pick on the agar adjacent to the worm (Figure 3A–B) or by freeze-thaw or low pH (Figure S2A,B). Hot pick-induced killing also caused fluorescent bursts in young adults of both sexes, and in larvae (Figures 3A–B, S2C; Video S2), and in the nematodes *C. briggsae* and *Pristionchus pacificus* (Figures 3A, S2D–E). In both aged and killed worms, fluorescence distribution also changed during death, from punctate to diffuse, and eventually spread from the intestine to other tissues (Figures 2B, S3). We named this phenomenon *death fluorescence* (DF).

Next we characterized the spatiotemporal dynamics of DF in *C. elegans*, as a potential marker of cellular and organismal death. DF typically originates in the anterior-most cells of the intestine (the int1 cells). It then spreads rapidly along the intestine in an anterior to posterior wave (Figures 3C–D, S3). In adults a second focus of fluorescence sometimes appears in the mid-body (Figure S3). When a hot pick was applied to the animals' tails (rather than near the head), DF initially only arose locally and did not spread from posterior to anterior (Figure 3D) but only, eventually, from anterior to posterior (unpublished data). This suggests that the anterior intestine represents an organismal weak point in *C. elegans*, where a local crisis in homeostasis can trigger a DF wave.

Several types of autofluorescence with different spectral properties have been described in *C. elegans* [29],[33],[34] (see Figure S4A for an overview of worm fluorescence). Using a more sensitive detection system [33], we examined the dynamics of blue, green, and red fluorescence over life and aging-induced death. Again, no significant age-increase in blue fluorescence was seen (Table S1). The much weaker green and red fluorescence did increase significantly with age (Figure S5; Table S1), and all three forms of fluorescence increased during death (Figure S5; Table S1). These results imply the presence of multiple fluorophores in *C. elegans*.

### Blue Fluorescence Emitted by Anthranilic Acid Glucosyl Esters

We then investigated the chemical identity of the blue fluorophore, using *glo-1(zu437)* (gut granule loss 1) mutants that lack gut granules [19]. *glo-1* animals showed little blue fluorescence either during life or death due to aging or thermal injury (Figures S4B, S6A–B), implying that gut granule fluorescence and DF have a common origin. Blue fluorescence was present in aqueous extracts of N2 (wild type) worm homogenates. HPLC analysis revealed one major peak with fluorescence at λ_ex_/λ_em_ 340/430 nm in N2 but not *glo-1* extracts (Figure S6C–D). We therefore used *glo-1* mutants as a negative control for chemical identification of the blue fluorophore, using 2D NMR-based comparative metabolomics [35]. This approach allows identification of compounds whose production depends on a specific genetic background without extensive chromatographic fractionation.

**Figure 4:**
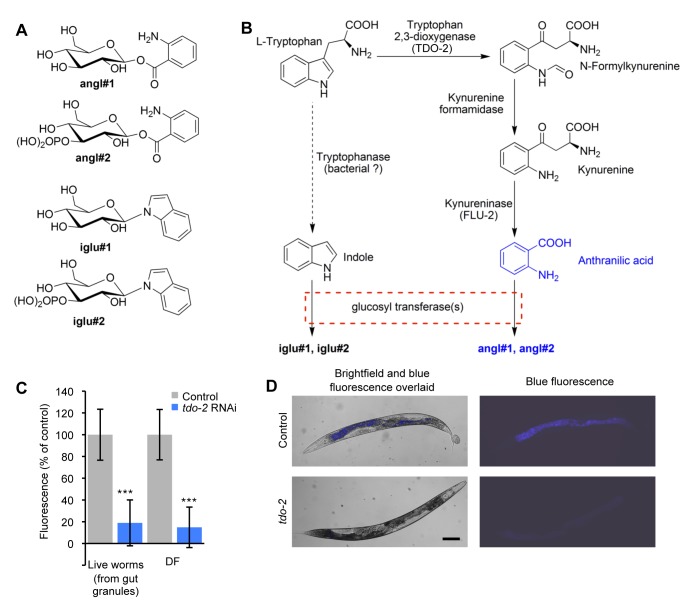
DF is generated by the kynurenine pathway. (A) Compounds down-regulated in *glo-1(zu437)* background. (B) Anthranilic acid (AA) is derived from tryptophan via the kynurenine pathway. angl#1 and angl#2 may result from action of glucosyltransferases. (C and D) RNAi of putative tryptophan 2,3-dioxygenase (C28H8.11) significantly reduces both gut granule and DF. Bar, 100 µm, error bars ± SD. Negative fluorescence shown by error bars are due to normalization via subtraction of background fluorescence.

Comparison of 2D NMR spectra acquired for the N2 and *glo-1* extracts revealed several groups of signals that were much reduced or absent in the *glo-1* spectra (Figure S7). The most differentially expressed compounds were the anthranilic acid glucosyl ester (angl#1 in Figure 4A) and N-glucosyl indole (iglu#1) and their corresponding 3′-phosphorylated compounds (angl#2 and iglu#2). N2 but not *glo-1* extracts also contained smaller quantities of free anthranilic acid. These structural assignments were confirmed via high-resolution mass spectrometry and synthesis of authentic samples of anthranilic acid glucosyl ester and N-glucosylindole (Tables S2, S3, S4). Moreover, fluorescence spectra for angl#1 and worm blue fluorescence were alike (Figure S8).

Anthranilic acid (AA) is synthesized from L-tryptophan (Trp) by action of the kynurenine pathway (Figure 4B), and has previously been observed in *C. elegans* [36],[37], but neither angl#1 nor angl#2 have previously been identified in animals. AA derivatives show fluorescence at λ_ex_/λ_em_ 340/430 nm [38]. The HPLC retention times of these AA derivatives matches those detected in the initial HPLC analysis of the N2 extract (Table S4). The indole glucosides iglu#1 and iglu#2, also not previously reported in animals, did not emit blue fluorescence (unpublished data).

To verify the identity of the blue fluorophore, we next used a genetic approach. The first step in the conversion of Trp to AA is catalyzed by tryptophan 2,3-dioxygenase (TDO). The *C. elegans* gene *tdo-2* (C28H8.11) encodes a putative TDO (Figure 4B) [36]. *tdo-2*(RNAi) suppressed both gut granule blue fluorescence and DF (freeze-thaw) (Figure 4C–D). RNAi or mutation of *flu-2* (kynureninase) also reduced DF, while inactivating kynurenine 3-monooxygenase by *kmo-1*(RNAi) or *flu-1* mutation increased DF, all as predicted (Figure S9). Both *tdo-2*(RNAi) and mutation of *flu-2* greatly reduce AA levels [37]. We also tested whether exogenous AA is sufficient to cause blue gut granule fluorescence. *tdo-2*(RNAi) worms were incubated in a range of solutions of synthetic anthranilic acid (Sigma). Incubation with 5 mM AA restored gut granule fluorescence to a wild-type level (Figure S10A–B). We conclude that gut granule fluorescence and DF emanate from AA.

### DF Does Not Require Increased Fluorophore Concentration

Why do fluorescence levels increase at death? One possibility is that AA levels increase at the point of death. To test this we compared DF in *tdo-2*(RNAi) worms with restored gut granule fluorescence and in L4440-treated control worms. Interestingly, the magnitude of DF was not reduced in the *tdo-2*(RNAi) worms (Figure S10C–D). This strongly implies that the DF burst is not the result of increased AA levels.

An alternative possibility is that concentration of AA within gut granules results in quenching of fluorescence. To probe this idea we employed the dye uranin, whose green fluorescence is partially quenched at low pH [39]. Treatment of wild-type worms with uranin led to punctate green fluorescence in the intestine that co-localized with blue gut granule fluorescence (Figure S11A). Thus, uranin accumulates within gut granules. Killing after uranin treatment caused a burst of green fluorescence in wild-type worms, but not *glo-1(zu437)* mutants without gut granules (Figure S11B–C). This supports the view that the burst of fluorescence at death is caused by dequenching of AA and uranin fluorescence due to increased pH upon release from the acidic milieu of the gut granules. It also shows that uranin is an excellent marker for loss of integrity of membranes bounding acidic compartments (e.g., lysosomes and lysosome-like organelles).

### DF Is Generated by Necrotic Cell Death

**Figure 5:**
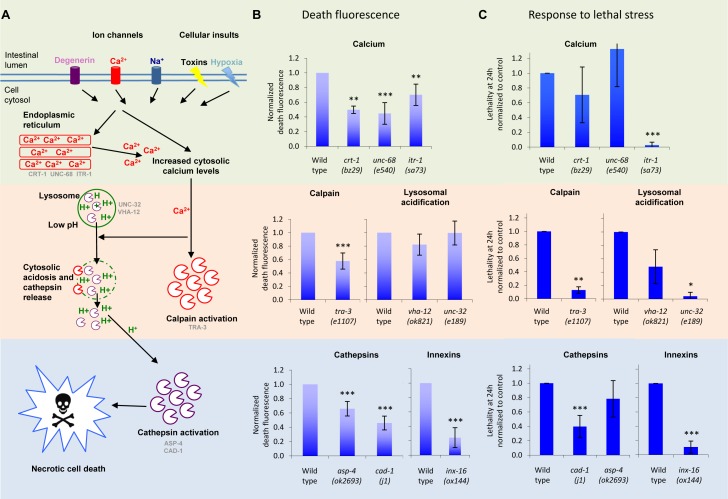
Inhibition of necrosis pathway reduces DF. (A) The calpain-cathepsin necrosis cascade. (B) Effects of inhibition of necrosis on DF, in ER calcium mutants, a calpain mutant, lysosomal acidosis mutants, cathepsin mutants, and an innexin mutant. Death was induced by freeze-thaw in young adults. Plots in (B) and (C) show mean ± SD, *p<0.05, **p<0.01 and ***p<0.001. Statistics: Wild type, *N* = 7 independent assays (different days), 50 worms/assay *n* = 350; *asp-4*, *N* = 7, *n* = 350, *p*<0.001; *crt-1*, *N* = 3, *n* = 150, *p*<0.01; *unc-68*, *N* = 6, *n* = 300, *p*<0.001; *itr-1*, *N* = 6, *n* = 300, *p*<0.01; *tra-3*, *N* = 6, *n* = 300, *p*<0.001; *vha-12*, *N* = 7, *n* = 350, *p*>0.05; *unc-32*, *N* = 6, *n* = 300, *p*>0.05; *cad-1*, *N* = 7, *n* = 350, *p*<0.001; *inx-16*, *N* = 6, *n* = 300, *p*<0.001. (C) Most necrosis mutants are significantly more resistant to death induced by osmotic stress (500 mM NaCl). Wild type, *N* = 6, *n* = 300; *asp-4*, *N* = 7, *n* = 350, *p*>0.05; *crt-1*, *N* = 5, *n* = 250, *p*>0.05; *unc-68*, *N* = 2, *n* = 150, *p*>0.05; *itr-1*, *N* = 3, *n* = 300, *p*<0.001; *tra-3*, *N* = 3, *n* = 100, *p*<0.01; *vha-12*, *N* = 3, *n* = 150, *p*>0.05; *unc-32*, *N* = 2, *n* = 100, *p*<0.05; *cad-1*, *N* = 6, *n* = 300, *p*<0.001; *inx-16*, *N* = 4, *n* = 200, *p*<0.001.

The presence of blue fluorescence within lysosome-related organelles (i.e., gut granules) and the central role of LMP in multiple instances of necrotic cell death suggested that DF might be generated by necrosis. If correct, then inhibition of necrosis might be expected to suppress DF. To test this we used freeze-thaw induced death, which is convenient for rapid and accurate quantitation of DF. The calpain-cathepsin necrotic cascade (Figure 5A) is involved in *C. elegans* neurodegeneration [15]. Neuronal necrosis requires Ca^2+^ release from ER stores. Mutations in the ryanodine and inositol-1,4,5-triphosophate receptors, and the ER Ca^2+^ binding protein calreticulin all suppress neuronal necrotic cell death [17],[40]. Each of these mutations, *unc-68(e540)*, *itr-1(sa73)* and *crt-1(bz29)*, respectively, also significantly reduced DF (Figure 5B). During cellular necrosis, increased intracellular Ca^2+^ can activate calpains (Ca^2+^-dependent cysteine proteases). The calpain TRA-3 is required for neuronal necrotic cell death in *C. elegans* [41]. *tra-3(e1107)* reduced DF (Figure 5B).

The necrosis cascade requires lysosomal lysis for cytosolic acidification and cathepsin release. In worms, the vacuolar proton-translocating ATPase (V-ATPase), which mediates lysosomal acidification, is required for necrosis [18]. We therefore tested two hypomorphic V-ATPase mutants, *vha-12(ok821)* and *unc-32(e189)*, but these did not significantly reduce DF (Figure 5B). Finally, we asked whether cathepsins promote DF. *cad-1(j1)* mutants have 10%–20% of wild-type cathepsin D activity [42] and *asp-4* encodes an aspartyl protease: both genes are required for necrosis [15],[18]. Again, both *cad-1(j1)* and *asp-4(ok2693)* reduced DF (Figure 5B).

*ced-3*, *ced-4*, and *ced-9* are required for apoptosis in worms [43]. To test whether apoptotic cell death machinery contributes to DF, we examined DF in killed *ced-3(n717)*, *ced-4(n1162)*, and *ced-9(n1950)* mutants. *ced-9* mutants showed no decrease in DF, *ced-4* mutants only a slight decrease, and *ced-3* actually showed an increase in DF (Figure S12A). In other negative controls (*ftn-1*, *mdl-1*, and *rol-6*) no effects on DF levels were seen either (Figure S12B). Thus, mutations that inhibit ER Ca^2+^ release, and calpain and cathepsin activity both inhibit necrosis and lower DF. We conclude that attenuation of elements of necrosis reduces DF. This suggests not only that cellular necrosis generates DF, but also that cellular necrosis occurs during organismal death. This in turn suggests the possibility that necrotic cell death contributes to organismal death.

### Organismal Propagation of DF by Calcium Signaling

The spread of DF through the intestine is reminiscent of Ca^2+^ wave transmission in the intestine during the defecation cycle [44]. Innexins (invertebrate gap junction proteins) are required for the defecation cycle as they create Ca^2+^ channels between adjacent intestinal cells, and the innexin INX-16 is required for Ca^2+^ transmission during defecation [44]. We asked whether Ca^2+^ signaling might play a role in DF wave propagation.

**Figure 6:**
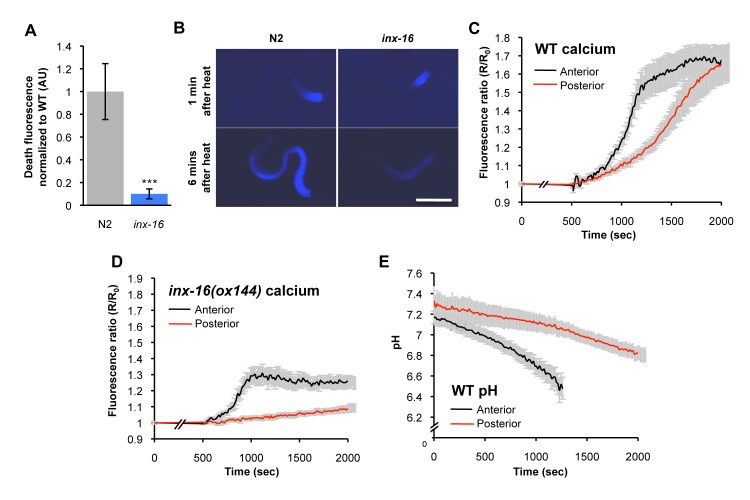
The spread of DF is dependent on calcium, and is accompanied by cytosolic acidosis. (A and B) *inx-16(ox144)* reduces DF and prevents its propagation (death induced using heated wire). (C–E) Ca^2+^ levels and pH in the intestine of worms killed by oxidative stress (*t*-BOOH). (C) *In vivo* Ca^2+^ levels rise at death in the anterior intestine prior to the posterior intestine, consistent with an anterior to posterior Ca^2+^ wave. Mean ± SD. (D) The Ca^2+^ reporter expressed in an *inx-16(ox144)* strain confirms that Ca^2+^, like DF, rises in the anterior but does not spread. Mean ± SD. (E) *In vivo* pH decreases at death from pH ∼7.35 to ∼6.6 in the anterior intestine prior to the posterior intestine, consistent with an anterior to posterior wave of cytosolic acidosis. Mean ± SD. In (C–E), “anterior” indicates the int1 and int2 anterior intestinal cells, and “posterior” the int9 posterior intestinal cells.

Upon being killed (by freeze-thaw), *inx-16(ox144)* mutants showed reduced DF levels, and (by a hot pick) a failure in DF wave propagation (Figures 6A–B, S13A; Videos S3, S4). Note that DF dynamics appear largely independent of the mode of killing (Figures 3, S2). Moreover, an intestinally expressed calcium reporter revealed increased Ca^2+^ levels during death (by oxidative stress; *t*-BOOH) (Figure 6C). This increase occurred first in the anterior and then in the posterior intestine (Figures 6C, S13B; Video S5), consistent with a wave of Ca^2+^ influx during death. The death-induced Ca^2+^ wave was blocked by *inx-16(ox144)*, as observed for DF (Figure 6D). Thus, Ca^2+^ signaling is required for, and precedes, the spread of DF. This suggests that during death an anterior to posterior wave of Ca^2+^ influx drives a wave of necrosis that leads to DF.

Cytosolic acidosis and LMP also typically occur during necrotic cell death, and we therefore asked whether they accompany DF. To test for cytosolic acidosis, we used an intestinally expressed pH-sensitive reporter, p*nhx-2*::pHluorin [45],[46]. Upon killing (with *t*-BOOH), pH in the intestinal cytosol dropped from ∼pH 7.4 to 6.6 (Figure 6E, Video S6) and, again, cytosolic acidosis occurred first in the anterior intestine before spreading to the posterior (Figures 6E, S13C–D).

**Figure 7:**
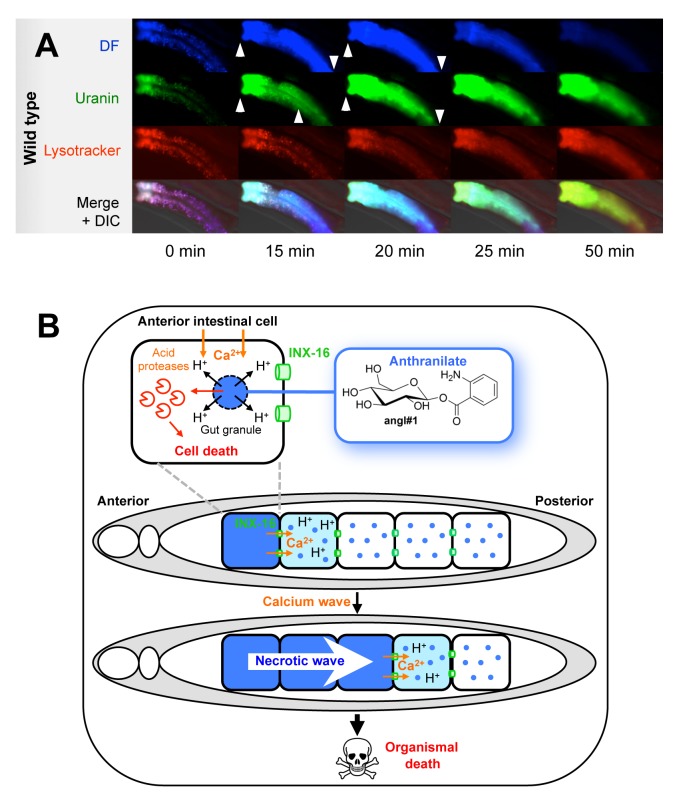
Stress resistance of necrosis mutants suggest model for organismal death. (A) Wave of blue fluorescence is accompanied by loss of lysosomal membrane integrity upon killing with *t*-BOOH. The quenching of uranin fluorescence at low pH makes it an excellent marker for loss of membrane integrity in lysosome-related organelles. (B) Working model of DF and systemic necrosis during organismal death in *C. elegans*. Unidentified factors (possibly calcium influx) trigger an initial necrotic event, typically in the anterior intestinal cells. This necrotic event includes cytosolic acidosis and LMP, which is associated with a burst of blue fluorescence from anthranilic acid glucosyl esters, which render visible the occurrence of necrosis. Necrosis in the anterior cell leads to calcium influx into the neighboring cells via INX-16 channels, triggering further necrotic events, and a cascade of necrosis along the intestine. In stress-induced death, but not aging-induced death, systemic necrosis can detectably hasten organismal death. However, it also seems likely that necrotic destruction of the intestine, a major organ within *C. elegans*, contributes to organismal death in senescent nematodes.

Next we used uranin (green) and the lysosomotropic dye lysotracker (red) to examine gut granule membrane integrity during organismal death. Killing (with *t*-BOOH) resulted in a burst of uranin fluorescence, and a loss of punctate green and red fluorescence that coincided with DF (Figure 7A). These changes were inhibited by *inx-16(ox144)* (Figure S14). The punctate red staining took slightly longer to decay than the green, likely reflecting residual staining of lysosomal membranes with lysotracker but not uranin. Thus, both cytosolic acidosis and LMP occur in intestinal cells at death. This provides further evidence that DF is generated by necrotic cell death.

### Attenuation of Necrosis Protects Against Stress-Induced Death

If systemic necrosis contributes to organismal death, then its inhibition should prevent death. To test this we examined the effect of inhibiting necrosis on death induced by aging or stress. In most cases, inhibiting necrosis did not prevent death due to aging: necrosis mutants were either normal lived or short lived (Figure S15). The exception was *inx-16*, which was long lived; however, the slow growth and starved appearance of this strain suggests that its longevity may be caused by dietary restriction.

By contrast, inhibition of each point in the necrosis pathway (calcium release, calpains, lysosomal acidification, cathepsins, and innexins) was able to delay death induced by lethal osmotic stress (Figure 5C), although not all mutants showed resistance. Most necrosis mutants also showed resistance to lethal thermal stress (Figure S16). Moreover, it was previously reported that inhibition of necrosis can delay infection-induced death [47]. However, necrosis mutants showed little protection against death induced by oxidative stress (*t*-BOOH) (unpublished data). These findings suggest that some stressors cause death in *C. elegans* by triggering systemic necrosis.

One possibility is that the release of AA from gut granules stimulates intestinal necrosis. To test this we first examined the effect of removing AA by *tdo-2*(RNAi) on resistance to heat stress, and found that resistance was increased (Figure S17A–C). However, it was previously shown that *tdo-2*(RNAi) enhances proteostasis and lifespan by increasing Trp levels [37]. To test whether *tdo-2*(RNAi) protects against heat stress by reducing AA, we asked if replenishment in *tdo-2*(RNAi) worms would suppress their stress resistance, but it did not (Figure S17C). Moreover, AA supplementation of N2 worms did not reduce heat stress resistance. Also in a range of mutants with altered AA levels there was no correlation with resistance to either thermal or osmotic stress (Figure S17D–E). Thus, heat stress resistance resulting from *tdo-2*(RNAi) is not caused by reduced AA but may instead reflect increased Trp levels. These results imply that DF does not promote intestinal cell necrosis but, rather, is a bystander phenomenon (or epiphenomenon).

We also tested the effect of insulin/IGF-1 signaling on DF. Mutation of the *daf-2* insulin/IGF-1 receptor increases lifespan, and this effect requires the *daf-16* FoxO transcription factor [48]. In both freeze-thaw- and aging-induced death, the mutation *daf-2(e1370)* markedly reduced DF (Figure S18). This could imply that systemic necrosis is attenuated in *daf-2* mutants. *daf-16(mgDf50)* modestly increased DF during death from old age only (Figure S18).

## Discussion

In this study, we have shown that conserved mechanisms underpinning neuronal necrosis can also contribute to organismal death. In so doing we provide the first insights into the last biological events in the life history of *C. elegans*: those leading to its final demise. Identification of an endogenous fluorescent marker of death led us to discover a calcium-generated wave of necrotic cell death that occurs during, and can contribute to, organismal death (Figure 7B). We have also chemically defined the source of the endogenous blue fluorescence that is a salient characteristic of *C. elegans*.

### Intercellular Propagation of Necrosis in *C. elegans* Death and Mammalian Neurodegeneration

Evidence presented here implies that during death in *C. elegans*, the intestine, the largest somatic organ, undergoes a stereotyped process of self-destruction involving an intra- and intercellular cascade of cellular necrosis. The mechanisms involved are similar to those active in the propagation of cellular necrosis in mammals. In worms, necrotic propagation requires the innexin INX-16, while in mammals connexin (mammalian gap junction proteins) inactivation reduces ischemia-induced neurodegeneration [10]. Thus, the *C. elegans* intestine is a potential new model for understanding the propagation of necrotic cell death, and its prevention.

Previous studies of the cellular necrosis pathway have largely focused on neurodegeneration, in mammals and *C. elegans*. Our findings imply similar action of this pathway in the worm intestine. However, generation of DF appears to be restricted to the intestine, and is not detectable in necrotic *mec-4(d)* neurons (unpublished data).

Our results imply that intestinal self-destruction by systemic necrosis occurs during both stress- and aging-induced death. However, only in stress-induced death did inhibition of systemic necrosis prevent death. This suggests that while lethal stress causes death by inducing systemic necrosis, aging causes death by a number of processes acting in parallel, likely including systemic necrosis (given that it destroys a major organ). Here there are potential parallels in human aging: estimations of the likely upper limits of human longevity have calculated that removal of a major age-related disease (e.g., cardiovascular disease, cancer) would cause only small increases in lifespan [49]. This is because multiple pathologies act in parallel to increase age-related mortality.

A feature of intestinal necrosis is its origin in the anterior int1 cells. This suggests that the unusual vulnerability of these cells to necrotic death might represent a breaking point within organismal homeostasis; analogously, in humans localized failure (e.g., in the heart or kidneys) can cause rapid organismal death. The existence of an anterior to posterior (A-P) Ca^2+^ wave is unexpected, given that the defecation-associated Ca^2+^ wave previously characterized in the intestine flows in the opposite direction, from posterior to anterior [44]. How the A-P Ca^2+^ wave is specified is unknown. One possibility is that extracellular Ca^2+^ levels are elevated near the anterior intestine, creating vulnerability to necrosis [50].

### *C. elegans* Blue Fluorescence Is Not Lipofuscin

In this study we have defined a new phenomenon, death fluorescence, which may be useful in future as a marker of death in lifespan assays. While DF means that blue fluorescence cannot be used as a biomarker of aging we confirm that red fluorescence does increase with age. Moreover, *tdo-2*(RNAi) can be used to abrogate blue intestinal fluorescence to aid the viewing of expression of intestinally expressed fluorescent reporters.

The anthranilic acid glucosyl ester angl#1 and its corresponding 3′-phosphorylated derivative angl#2 account for both death and gut granule-associated fluorescence. *glo-1* mutants lack both forms of fluorescence and AA derivatives, and inhibition of the kynurenine pathway blocks both forms of fluorescence, establishing that this blue fluorescence is not lipofuscin. Whether lipofuscin accumulation occurs during aging in *C. elegans* remains an open question. However, our finding that blue fluorescence is not lipofuscin removes one reason for believing that aging in *C. elegans* is caused by accumulation of stochastic molecular damage. The kynurenine pathway that generates gut granule and DF is also involved in mammalian neurodegeneration, and has recently been shown to regulate protein folding homeostasis in *C. elegans* [37],[51].

During organismal death, AA fluorescence increases as a consequence of necrosis. That DF can occur in the absence of AA synthesis (Figure S10C–D) implies that the burst is not a consequence of synthesis of additional AA. One possibility is that AA fluorescence within gut granules is partially quenched, perhaps due to low pH and/or increased concentration. In this scenario, loss of gut granule membrane integrity causes rapid dequenching of AA fluorescence, leading to the burst. In a similar fashion, dequenching of uranin fluorescence upon gut granule permeabilization leads to a burst of green fluorescence (Figure S11). It remains to be investigated whether cellular necrosis in other organisms leads to increased AA fluorescence, but increases in blue fluorescence accompanying cell death have been reported—e.g., in budding yeast [52] and hepatocytes [53].

### Possible Functions of Anthranilic Acids and Systemic Necrosis in *C. elegans*

Despite their prominence, *C. elegans* gut granules are organelles whose function has yet to be established. The finding that they contain large quantities of AA further adds to the mystery. What is all this anthranilic acid for? Possibilities include protection against UV irradiation, or against pathogen invasion into the intestine. Notably, AA can be cytotoxic; for example, 3-hydroxyanthranilic acid can induce cell death in lymphocytes [54] and neurons [55], and AA can inhibit growth of bacterial pathogens (e.g., *Legionella pneumophila*) [56]. This might explain its presence in multiple species of soil nematodes.

The presence of a mechanism, systemic necrosis, that brings about organismal death in *C. elegans* raises questions about its evolutionary origin. Could such an organismal self-destruct mechanism serve as an adaptation? When food is limiting, gravid hermaphrodites typically die with multiple embryos in their uterus, which hatch internally and consume their mother's corpse (“bagging”). Potentially, this improves the mother's fitness by increasing survival of her genetically identical offspring [57]. One possibility, then, is that systemic necrosis enhances fitness by aiding efficient transfer of nutrients from mother to offspring during bagging. Alternatively, systemic necrosis may be the nonadaptive product of antagonist pleiotropy, or a quasi-program [58],[59]. By this view, elements of the necrosis cascade contribute to early life fitness, while systemic necrosis is an unselected, deleterious consequence of their action under lethal stress or as a result of aging.

## Materials and Methods

### Worm Maintenance and Strains

Standard *C. elegans* strain maintenance and genetic manipulations were used [60]. All strains were grown at 20°C on NGM plates seeded with *E. coli* OP50 as food source unless otherwise specified. N2 (Bristol) was the wild-type.

The necrosis mutants are as follows: ZB1028 *crt-1(bz29) V*, CB540 *unc-68(e540) V*, JT73 *itr-1(sa73) IV*, CB4027 *tra-3(e1107) eIs2137 IV*, CB189 *unc-32(e189) III*, RB938 *vha-12(ok821) X*, RB2035 *asp-4(ok2693) X*, and PJ1 *cad-1(j1) II*. The apoptosis mutants are as follows: MT1522 *ced-3(n717) IV*, MT4770 *ced-9(n1950) III*, and MT2547 *ced-4(n1162) III*. The strains used for calcium measurements are as follows: KWN190 *pha-1(e2123ts) III*, *him-5(e1490) V*; *rnyEx109* [pKT67 (P*nhx-2*::D3cpv); pCL1 (*pha-1*(+)], KWN26 *pha-1(e2123ts) III*; *him-5(e1490) V*; *rnyEx006* [pIA5nhx-2 (P*nhx-2*::pHluorin); pCL1(*pha-1*(+)]. The strains used for pH measurements are as follows: KWN385 *inx-16(ox144) I; pha-1(e2123ts) III*; *him-5(e1490) V rnyEx006* [pIA5*nhx-2* (P*nhx-2*::pHluorin); pCL1 (*pha-1*(+)]. Other: BA671 *spe-9(hc88) I*, CB1002 *flu-1(e1002) V*, CB1003 *flu-2(e1003)* X, GH10 *glo-1(zu437) X*, EG144 *inx-16(ox144)*, GA91 *ftn-1(ok3625) V*; GA1200 *mdl-1(tm311) X*, GA200 *wuEx41* [*rol-6(su1006)*], RB784 *nkat-1(ok566) X*, SJ4005 *zcIs4* [*hsp-4*::GFP].

### Oxidative Stress Assays

#### Hyperoxia treatment

Young N2 adults were placed in either 90% O_2_ or air at 25°C for 5 d on 10 µM FUdR (fluorodeoxyuridine), OP50 seeded NGM plates.

#### Iron treatment

We prepared 15 mM ferric ammonium citrate (FAC) plates as previously described [61]. Young N2 adults were placed on fresh NGM or FAC plates for 24 h prior to fluorescence and carbonyl measurements.

#### Heat shock treatment

L4 SJ4005 *zcIs4* [*hsp-4*::GFP] worms were placed at 30°C for 3 h or left at 20°C on OP50 seeded NGM plates prior to fluorescence measurement. Heat-shocked worms were allowed to recover for 15 min at 20°C before image capture.

#### Carbonyl measurement

Five-day-old worms were rinsed off plates, washed with M9 buffer, and stored at −75°C. Worm samples were homogenized using a Bioruptor (Cosmo Bio) in CelLytic (Sigma) lysis buffer containing protease inhibitors (Roche) and 40 mM DTT. The resulting homogenate was centrifuged at 20,000 rpm for 30 min at 4°C. The supernatant was collected and protein concentration determined by the Bradford method (Bio-Rad). Detection of carbonyl groups was performed with the OxyBlot oxidized protein detection kit (Chemicon International) as described previously [62] with minor modifications. Briefly, 20 µg of total extract proteins were derivatized before SDS-PAGE separation and electrotransfer. Modified proteins were revealed by antidinitrophenol antibodies. The total amount of signal was quantified by densitometric analysis using ImageQuant TL (GE Healthcare Europe). Three independent biological replicate worm cultures were assayed.

#### Blue fluorescence measurements

A minimum of 30 worms were individually photographed *in situ* on NGM plates (for details, see below).

### Microfluorimetry

Worms were either imaged *in situ* on NGM plates or anaesthetized on agar pads on glass slides. Images were acquired using an Orca digital camera (Hamamatsu) and a Leica DMRXA2 microscope. Blue fluorescence was observed through a DAPI filter cube (λ_ex_/λ_em_ 300–400 nm/410–510 nm) (ET DAPI, set 49000, Chroma). Green fluorescent protein (GFP) fluorescence was observed through a GFP filter, (λ_ex_/λ_em_ 450–490 nm/500–550 nm) (Endow GFP Bandpass, 41017 Chroma). Images were acquired using the application Volocity Acquisition (Improvision, Perkin-Elmer). Fluorescence was quantified by manually tracing around worm peripheries using an Intuos graphics tablet (Wacom), and measuring mean pixel density using Volocity Quantitation. Worm fluorescence was estimated as the mean pixel density of the worm image area minus the pixel density of the image background.

#### Time-lapse photography

Wild-type (N2) L4 hermaphrodites were cultured individually (one per plate) on NGM agar plates with *E. coli* OP50 and 10 µM FUdR [63]. Worms were individually photographed *in situ* on the plates every 1–2 d until death and beyond as described below. Nematodes were placed on ice for 10 min prior to photography to induce stillness. Nematode viability phenotype was scored daily as described [32]. Class C animals that move only their heads in response to touch usually die within 24 h. Upon identification, these animals were monitored using time-lapse photography (one image taken every 15 to 30 min) for the subsequent 12–24 h until after death.

#### Single-animal vermiculture

This was performed as previously described [33]. Fluorescence was measured under both DAPI and GFP filters, as before, and under a TRITC filter (λ_ex_/λ_em_ 530–560 nm/585–645 nm, TRITC 41002b, Chroma) (Figure S4A).

### *C. elegans* Killing Assays and DF Measurements

Worms were killed in three ways, detailed below, all of which result in bursts of blue fluorescence of similar magnitude. Heat killing was used to observe DF dynamics in individual animals *in situ* on NGM plates. This approach allows lethal stress to be applied near the head or tail, and allows observation of spatial changes in fluorescence, but is relatively difficult to quantitate. Killing by oxidative stress was used for higher resolution microscopy for which it was necessary to view worms under cover slips. Freeze-thaw assays of worms in microtitre plates were used for accurate quantitation of DF to compare genotypes cohorts of worms, and for acquisition of whole spectrum excitation/emission scans.

#### Heat killing

Here fluorescence of individual animals was measured *in situ* on NGM plates after killing with a heated worm pick, fitted with a platinum wire (0.3 mm diameter). The wire was flame heated immediately prior to use, and then placed briefly (<1 s) on the agar immediately adjacent to the worm (∼0.1–0.2 mm away), usually near the head end.

#### Exposure to *tert*-butylhydroperoxide

Worms were exposed to 0.726 M (or 7%) *t*-BOOH, diluted in M9, for 5 min. Following exposure, worms were moved to a 2% agarose pad for time-lapse imaging. Worms were imaged at 100× magnification, on a Nikon Eclipse TE2000-U inverted fluorescence microscope quipped with an excitation monochromator (Polychrome IV, TILL Photonics) and a high-speed monochromatic camera (Sensi-Cam, Cooke). Fluorescence emissions were monitored at 460 nm after 4 ms excitation at 360 nm. Images were captured every 10 s. Image capture and quantitative measurements were performed using the TILLvisION software package (TILL Photonics). Traces were plotted in Microsoft Excel.

#### Freeze-thaw

We put 45 or 50 1-d adults worms per condition in 150 µL M9 plus 0.2% levamisole in a V-shaped 96-well plate (Greiner Bio-One, Frickenhausen, Germany). The plate was then centrifuged at 1,500 rpm for 1 min and frozen at −80°C for 15 min. The plate was then incubated at 42°C in an Infinite 200 PRO plate-reader running Magellan software (Tecan Group Ltd., Switzerland) and blue fluorescence measurements were acquired every 30 s for 30 min and for each well, using λ_ex_/λ_em_ 340±9/435±20 nm. A peak in fluorescence intensity occurred at ∼7–10 min marking the release of gut granule AA into the cytosol, and giving a readout of total AA fluorescence. Relative AA fluorescence was calculated as the difference between the initial minimum and subsequent peak fluorescence intensities for each well, normalized to the N2 control. To account for variation in worm size between conditions (genetic background or RNAi treatment), relative AA fluorescence was then divided by a size factor. This size factor was obtained by imaging 30 worms by DIC microscopy at 10× for each condition, measuring intestinal cross-sectional area and normalizing it to the control (N2) intestinal cross-sectional area.

### Whole-Spectrum Excitation Emission Scans

Synchronous populations of L4 animals were transferred to NGM plates seeded with *E. coli* OP50 and containing 50 µM FUdR (24°C). One-day-old adults were then rinsed off the plates and washed using S buffer. For each strain, three replicate aliquots of 100 µl worm suspensions were loaded in black microtiter plates (Greiner). Aliquots resulted in ∼1 mg alkali-extracted protein, determined by standard BCA assay (ThermoScientific). The 10-nm step fluorescence emission spectra of living worm suspensions were measured upon excitation at 250–450 nm (10 nm intervals) using a microplate reader (Spectramax Gemini XS, Molecular Devices). Worms were then killed by freeze-thaw and fluorescence measurements repeated. All data shown are averages of three technical replicates, corrected by a blank measurement, and normalized by protein content of the worm suspensions.

### Compound Identification: Analytical Instrumentation and Procedures

#### NMR spectroscopy

Differential analysis by 2D-NMR spectroscopy (DANS) is a two-dimensional analysis of metabolomes derived from different genotypic backgrounds. This method accelerates the identification of genome-specific compounds because it does not require extensive chromatographic fractionation. In traditional structural elucidation schemes, chromatographic fractionation is a major cause of compound loss due to degradation or decomposition, and so can significantly hamper the speed of identification [64]. Several recent examples have demonstrated the utility of a specific type of 2D NMR spectrum (double quantum filtered correlation spectroscopy, or dqfCOSY) for compound identification via DANS in *C. elegans* [35],[65].

NMR spectra were recorded on a Varian INOVA 600 NMR (600 MHz for 1H, 151 MHz for 13C) and INOVA 400 NMR (400 MHz for 1H, 100 MHz for 13C) instruments. Nongradient phase-cycled dqfCOSY spectra were acquired using the following parameters: 0.6 s acquisition time, 500–900 complex increments, 8–32 scans per increment. Gradient and nongradient HSQC[AD], HMQC, and HMBC[AD] spectra were acquired with these parameters: 0.25 s acquisition time, 300–600 increments, 8–32 scans per increment. ^1^H,^13^C-HMBC spectra were optimized for J_H,C_ = 6 Hz. Susceptibility-matched NMR tubes (Shigemi) were used for sample amounts smaller than 2 mg. NMR spectra were processed using Varian VNMR, MestreLabs MestReC, and Mnova software packages.

#### HPLC-MS

This was performed using an Agilent 1100 Series HPLC system equipped with a Varian Pursuit XRs-3-C18 column (4.6×250 mm, 5 µm particle diameter) connected to a Quattro II spectrometer (Micromass/Waters). A water (containing 0.1% acetic acid) – acetonitrile (also containing 0.1% acetic acid) solvent gradient was used at a flow rate of 0.7 ml/min: acetonitrile at 5% for 15 min increased to 35% over 20 min, subsequently to 95% over 5 min, and continued at 95% for 6 min. Metabolite extracts were analyzed by positive and negative electrospray ionization-MS. High-resolution HPLC-MS was performed using a Waters nanoACQUITY UPLC System equipped with a Waters Acquity UPLC HSS C-18 column (2.1×100 mm, 1.8 µm particle diameter) connected to a Xevo G2 QTof Mass Spectrometer.

#### Sample preparation

Whole worm sonicates were prepared in dH_2_O from large heterogeneous populations grown on NGM plates. Sonication was performed in repeated cycles of 1 min followed by a 30 s pause for cooling in a “Bioruptor” water bath sonicator (Diagenode) until samples were homogenously clouded. Samples were spun down at 4°C at 14,000 rpm for 15 min, and the supernatant extracted. Cold 100% ethanol was added in a 1∶9 sample∶ethanol ratio and left at −20°C overnight. Samples were spun down again as above. The remaining supernatant was taken. Samples were then evaporated in vacuum at room temperature. For NMR-spectroscopic analysis, the residues were dissolved in 200–600 µL of methanol-d4. Following NMR-spectroscopic analysis, samples were evaporated to dryness, resuspended in 500 µL methanol, centrifuged, and 1–30 µL aliquots were used for HPLC-MS analysis.

### Compound Syntheses

#### Synthesis of angl#1 (β-D-glucosyl anthranilic acid ester)

Adapting a previously described procedure [66], a catalytic amount (∼1 mg, 0.025 mmol) of sodium hydroxide was added to a solution of 75 mg (0.462 mmol) of isatoic anhydride (Sigma-Aldrich, I12808) and 250 mg (0.462 mmol) of 2,3,4,6-tetra-O-benzyl-D-glucopyranose (Sigma-Aldrich, 86730) in 4 mL dioxane. The reaction mixture was stirred and heated gradually until moderate evolution of CO_2_ occurred (75–90°C). This temperature was maintained until gas evolution ceased (2 h) and the mixture was cooled and diluted with 6 ml of water. Crude 2,3,4,6-tetra-O-benzyl-D-glucosyl anthranilic acid ester, containing both the a and b isomers, separated out as an immiscible oil and was extracted with methylene chloride (yield 255 mg, 84%). To deprotect the glucose moiety, this mixture of products was dissolved in ethyl acetate (10 ml) to which 10% palladium on activated carbon (28 mg) was added, and the resulting suspension was stirred under a hydrogen atmosphere for 24 h. Subsequently, the reaction vessel was flushed with argon, and the reaction mixture was filtered over Celite and the filtrate evaporated to dryness *in vacuo*. A 10 mg sample of pure angl#1 was isolated from the mixture of α- and β-isomers by silica gel column chromatography using 20% ethyl acetate and hexane.

#### Synthesis of iglu#1 (N-β-D-glucosyl indole)

A sample of iglu#1 was synthesized as described previously.

Structural assignments were confirmed via high-resolution MS and synthesis of authentic samples of anthranilic acid glucosyl ester and N-glucosylindole (Table S2, S3, S4) [66],[67].

#### Compound names

All newly identified *C. elegans* metabolites were named using their four letter “SMID”s (Small Molecule IDentifiers) (e.g., “iglu#2” or “angl#1”). The SMID database (www.smid-db.org) is an electronic resource maintained by Frank Schroeder and Lukas Mueller at the Boyce Thompson Institute in collaboration with Paul Sternberg and WormBase (www.wormbase.org). This database catalogues *C. elegans* small molecules, assigns a unique four-letter SMID, and for each compound lists other names and abbreviations used in the literature.

### Anthranilic Acid Supplementation

A 55 mM stock of anthranilic acid (Sigma) was prepared by dissolving AA solid in PBS at 55°C and then diluted further in PBS. Worms were incubated in AA solution for 3 h at 20°C in a 96-well microtitre plate with constant shaking.

### Uranin Staining

#### Uranin

100 µl of 20 mg/ml uranin (fluorescein sodium salt, Sigma Aldrich, Germany) was added topically to *E. coli* OP50-seeded NGM plates, and left to dry. Young adult N2 worms were placed on the stained plates for 3 h and then transferred to OP50 plates without uranin, and destained for 45 min. Fluorescence was measured using microscopy as described above, with an L5 FITC filter (λ_ex_/λ_em_ 460–500/512–542 nm) with a 1,500 ms exposure time.

For plate reader assays, 50 1-d adult worms under each condition were transferred into wells containing 150 µl PBS. Worms were washed 3× with fresh PBS. Finally, 100 µl PBS was replaced with 100 µl pure *t*-BOOH. For the freeze-thaw assay, 100 µl of PBS was replaced by 100 µl 0.2% levamisole. Worms were centrifuged for 2 min at 2,000 rpm at room temperature. Blue and green fluorescence was measured simultaneously every 30 s in a plate reader (InfiniteM200, Tecan) using λ_ex/em_ 340/410 nm and 490/520 nm, respectively.

### Blue Fluorescence, Lysotracker, and Uranin Co-localization Assays

L4 or 1-d-old adult worms were incubated for 2 h in 475 µL M9 plus 10 µL Lysotracker Red DND-99 (Life Technologies, USA) plus 15 µL 20 mg/mL uranin. Worms were then washed 5× in 1 mL M9 and left to feed on OP50-seeded NGM plates for 30–60 min. They were then placed on a 2.5% agarose pad prepared on a glass slide between two cover slip spacers (to avoid squashing of adult worms between the pad and the cover slip), and mounted in 0.2% levamisole under a cover slip. Worms were then imaged under at 100× magnification on a DM RXA2 upright microscope (Leica, Germany) every 10 s for up to 1 h using Volocity software (PerkinElmer Inc., USA). We added 15 µL of Luperox TBH70X *tert*-butylhydroperoxide solution (Sigma Aldrich, Germany) using a pipette, assuring even dispersal of the liquid between the slide and the cover slip within the first minute of imaging. DF appeared within the first 15–30 min and the time-lapse acquisition was stopped once the blue fluorescence wave had propagated from head to tail.

### RNAi

The *flu-2*, *kmo-1*, *nkat-1*, and *tdo-2* clones were acquired from the Ahringer library, and the inserts confirmed by DNA sequencing. *E. coli* HT115 were transformed with the clone and fed to animals as described [68].

### *In Vivo* Measurements

#### Calcium

Calcium levels were visualized using the calcium indicator d3cpv14 [69],[70] expressed from the intestine-limited promoter Pnhx-2. Unrestrained, live worms were imaged on a NGM plate seeded with *E. coli* OP50 bacteria under 100× magnification, with dual fluorescence emissions at 480 nm and 535 nm captured in a single frame through an optical beam-splitter (Optical Insights) after a 50 ms exposure at 435 nm. Images were captured every 15 s with 2×2 binning. Changes in the pixel/pixel signal were determined as R/R_0_, where the emission ratio (R) is divided by the initial emission ratio (R0). Images were acquired as described above in the exposure to *t*-BOOH killing protocol.

***Y*** Worms were imaged at 100× magnification, with fluorescence emissions monitored at 535 nm following sequential 20 ms excitations at 410 nm and 470 nm. Images were captured every 15 s with 2×2 binning. Approximate pH values were estimated from fluorescence ratios using a Boltzmann equation based upon a high-potassium nigericin calibration curve, as previously described [71]. In Ca^2+^ and pH measurements, “anterior” indicates the int1 and int2 anterior intestinal cells, and “posterior” the int9 posterior intestinal cells.

### Lifespan Measurements

Lifespans of synchronized population cohorts were measured as previously described [72] at 20°C with 15 µM FUdR topically applied.

### Stress Resistance Assays

#### Heat stress

Unseeded NGM plates were prewarmed to 35°C for 1 h. Fifty 1-d-old adults per condition were then rapidly transferred onto warmed plates and incubated at 35°C. Percent survival was scored every 2 h for 12–14 h. Immobile worms were poked and tested for swimming ability upon addition of a drop of M9 medium: worms devoid of movement were scored as dead. Two to six biological replicates were performed.

#### Osmotic stress

On day 1, osmotic stress plates (NGM containing 500 mM NaCl) were prepared and allowed to dry overnight. On day 2, 50 L4 worms per condition (genetic background or RNAi-treatment) were placed onto *E. coli* OP50-seeded regular NGM plates, and osmotic stress plates were seeded with OP50. On day 3, 1-d-old young adults were transferred onto osmotic stress plates. After 24 h worms were transferred back to OP50-seeded regular NGM plates, allowed to recover for 15–30 min and percent survival scored. Death was scored as for heat stress (see above). Two to six biological replicates were performed.

### Statistical Analysis

Lifelong fluorescence measurements were acquired from individual animals. Data were normalized to time of each animal's death and average fluorescence level acquired at hours prior to death. Data measuring groups of animals used mean data, with Student's *t* tests performed to check for statistical significance. All mean data were repeated at least in triplicate. Lifespan and thermotolerance data were analyzed by log-rank for significance; osmotic stress by one-way ANOVA of means. All fluorescent images represent averages seen.

## Supporting Information

Figure S1 **Blue fluorescence levels do not increase with increased expression of an unfolded protein response (UPR) associated gene.** After exposure to elevated heat levels (30°C, 3 h), *hsp-4*::GFP levels rise significantly but blue fluorescence levels remain unaffected. Mean of three biological replicates, ± SD, *** *p*<0.001, Student's *t* test.
(PDF)

Figure S2 **DF is induced by different methods of killing.** (A) DF when *C. elegans* are killed by freeze-thaw. Mean of 3 biological replicates, 60 worms per trial, ± SD, *** *p*<0.001. (B) DF when *C. elegans* are killed by low pH (acetic acid, pH 3). Mean of 3 replicates, 30 worms per trial. (C) Representative images of DF in young adult wild-type males. (D, E) DF in other nematode species. Representative images of DF in *C. briggsae* and *P. pacificus*.
(PDF)

Figure S3 **DF in young adult hermaphrodite killed with a heated wire.** During DF the pattern of fluorescence changes from punctate to diffuse. Note the secondary focus of DF that appears in the mid-body region; these occur in a proportion of animals. A and P, anterior and posterior ends of intestine. Arrowheads, spread of DF from intestine to other tissues. Scale bar, 200 µm.
(PDF)

Figure S4 **Relationship between major forms of** ***C. elegans*** **fluorescence and filter sets used in this study.** This scheme shows a typical full excitation/emission fluorescence spectrum (aqueous homogenate of 12-d-old worms, redrawn from [21]), and their relationship to the specifications of the filter sets used here. B, blue peak, arising from anthranilic acid glucosyl esters. T, ultraviolet peak attributed to tryptophan. F_1_ and F_2_, green fluorescence peaks attributed to flavins [21]. (B) At death (freeze-thaw), a large DF peak at λ_ex_/λ_em_ 340/430 nm appears in wild type (N2), but not in *glo-1* animals, which lack gut granules. White patches are negative values.
(PDF)

Figure S5 **Other sources of fluorescence increase at death in** ***C. elegans*** **.** (A) Average levels of fluorescence normalized to time of death, viewed under red, blue, and green filter sets (λ_ex_/λ_em_, respectively, TRITC 546/600 nm, DAPI 350/460 nm, and GFP 470/525 nm). Different types of fluorescence showed different rates of increase with time. Forty-three individual animals were measured under each filter set. Shaded area, ±1 SD. Each plot is scaled on separate arbitrary units as absolute-terms comparisons are not possible. Note that rates of fluorescence accumulation and spatial distribution of the blue, red, and green fluorescence differ, consistent with the presence of distinct fluorophores. (B and C) Typical images of worms prior to death (B) and during death (C) in each fluorescence band, showing altered spatial distribution of the fluorescence.
(PDF)

Figure S6 ***glo-1*** **animals do not show gut granule or DF.** (A and B) *glo-1(zu347)* animals do not show an increase in blue fluorescence during aging (A) nor DF upon killing (B), making them a useful negative control for establishing the chemical source of gut granule fluorescence and DF. Mean ± SD. Note that the gradual increase in mean blue fluorescence in wild-type worms in (A) reflects an age increase in the proportion of dying worms. (C and D) HPLC chromatogram reveals four peaks present in N2 but not *glo-1* animals, marked by black arrows.
(PDF)

Figure S7 **Biochemical identification of DF constituents.** (A) 3.4–4.2 ppm region of the dqfCOSY spectrum (600 MHz, methanol-d4) of N2 worm extracts show cross-peaks representing several glucose moieties (black boxes). Crosspeaks at f2 = 4.12 ppm correspond to proton 3-H in angl#2 and show additional J-splitting due to coupling with ^31^P of the adjacent phosphate group (double bordered boxes). (B) 3.4–4.2 ppm region of the dqfCOSY spectrum (600 MHz, methanol-d4) of *glo-1(zu437)* worm extracts do not show cross-peaks representative of the glucose moieties present in N2 worm extracts (black boxes). (C) Section of the dqfCOSY spectrum (600 MHz, methanol-d4) of N2 worm extracts showing crosspeaks for the anomeric protons of four glucose units. Two of these (red boxes) belong to the indole glucosides (iglu#1, iglu#2) and the other two (blue boxes) are part of the anthranilic acid glucosides (angl#1, angl#2). (D) Section of the dqfCOSY spectrum (600 MHz, methanol-d4) of *glo-1* worm extracts corresponding to the section of the N2 dqfCOSY spectrum shown in (C). Cross-peaks representing indole glucosides (iglu#1, iglu#2) and anthranilic acid glucosides (angl#1, angl#2) are much weaker or completely absent.
(PDF)

Figure S8 **Fluorescence spectra for angl#1 and worm blue fluorescence are highly similar.** Also shown is fluorescence of unconjugated anthranilic acid at the same concentration as angl#1 (2 mM). Note that AA fluorescence is less similar to worm blue fluorescence than that of angl#1. The blue fluorescence at λ_ex_<270 nM in angl#1 and AA may be suppressed in the worm by the presence of other substances; we noted that addition of worm lysate to AA markedly reduced this fluorescence (unpublished data).
(PDF)

Figure S9 **Kynurenine pathway genes affect DF levels.** (A) The kynurenine pathway (upper portion). (B) Effects of RNAi of kynurenine pathway genes on DF. Worms were killed using freeze-thaw, and fluorescence measured in a plate reader. *tdo-2* and *flu-2* promote AA production, and here RNAi reduced DF, as expected. *kmo-1* and *nkat-1* convert kynurenine into compounds other than AA. Thus, *kmo-1*(RNAi) increased AA levels, likely by increasing kynurenine availability for AA synthesis. By contrast, *nkat-1*(RNAi) only marginally increased AA levels. This suggests that in wild type there is substantial conversion of kynurenine to 3-hydroxykynurenine but not kynurenic acid. The effect of *nkat-2(ok566)* (see C) is consistent with this. (C) Effects of mutation of kynurenine pathway genes on DF. *flu-2(e1003)* greatly reduced DF, to levels similar to those in *glo-1(zu437)* mutants that lack gut granules. The weaker effect on DF of *flu-2*(RNAi) in (B) may reflect incomplete abrogation of *flu-2* expression. It is likely that *flu-1 V* and *kmo-1 V* are the same gene, since mutation of *flu-1* greatly reduced activity levels of kynurenine 3-monooxygenase (kynurenine hydroxylase) [73]. Consistent with this, *flu-1(e1002)*, like *kmo-1*(RNAi), greatly increased AA levels.
(PDF)

Figure S10 Anthranilic acid (AA) supplementation rescues gut granule and DF. (A, B) Incubation of *tdo-2*(RNAi) worms in 5 mM AA leads to gut granule fluorescence similar to wild type. (A) Effect on gut granule fluorescence of incubation in a range of AA concentrations. 5 mM AA gives fluorescence levels similar to wild type. (B) Epifluorescence microscopy reveals restoration of gut granule fluorescence by incubation in 5 mM AA. (C, D) Incubation of *tdo-2*(RNAi) worms in 5 mM AA leads to DF similar to wild type. (C) Effects of *tdo-2*(RNAi) and AA supplementation on peak DF. (D) Similar kinetics of DF in control worms and *tdo-2*(RNAi) worms with AA-replenished gut granules, even though only the former can synthesize AA.
(PDF)

Figure S11 **Uranin treatment leads to green DF.** (A) Treatment with uranin leads to punctate green fluorescence that co-localizes with blue gut granule fluorescence. (B and C) Worms were incubated with uranin and then killed. (B) Killing with *t*-BOOH and (C) with freeze-thaw. Killing of uranin-treated worms led to a burst of green fluorescence in the wild type (N2) but not in a *glo-1(zu437)* mutant, which has no gut granules. Anthranilate and uranin fluorescence do not overlap: in the absence of uranin, there was no increase in λ_em_ 490 nm fluorescence at death, nor did the presence of uranin increase λ_em_ 340 nm fluorescence at death. For unknown reasons, uranin caused a slight decrease and delay in λ_em_ 340 nm fluorescence at death, in both forms of killing. Note that freeze-thaw killing caused substantial background λ_em_ 490 nm fluorescence.
(PDF)

Figure S12 **Mutants not predicted to have reduced necrosis do not show decreased DF.** These are negative controls for data shown in Figure 5. Graphs show relative increase of fluorescence at death, induced by freeze-thaw (60 young adult worms/strain/replicate). Mean ± SD, 3 biological replicates, **p<0.01 and ***p<0.001. (A) Apoptosis defective mutants. These results imply that apoptosis does not contribute to DF. *ced-3* mutants show significantly increased DF, perhaps reflecting inhibition of necrosis by wild-type *ced-3*. (B) Random selection of mutants unconnected to cell death, which do not show any significant change in DF. The mutations were in the genes *ftn-1*, *mdl-1*, and *rol-6* (encoding ferritin, the MAD-like transcription factor and a cuticular collagen, respectively). Selection of these mutants as negative controls was on the basis of lack of a known association with necrosis, and availability in the laboratory.
(PDF)

Figure S13 **Calcium spread and acidosis are correlated with DF.** (A) Loss of *inx-16* prevents the spread of DF. (B and C) Ca^2+^ levels and pH in the intestine of worms killed by oxidative stress (*t*-BOOH). (B) *In vivo* Ca^2+^ levels rise at death in the anterior intestine prior to the posterior intestine, consistent with an anterior to posterior Ca^2+^ wave. (C) *In vivo* intestinal pH decreases at death from pH ∼7.4 to ∼6.3, and this occurs more rapidly in the anterior than the posterior intestine, consistent with an anterior to posterior wave of cytosolic acidosis. Mean ± SD. Here “anterior” indicates the int1 and int2 anterior intestinal cells, “middle” the juxta-vulva region (∼int5), and “posterior” the int9 posterior intestinal cells. (D) Key to pH sensor in Video S6.
(PDF)

Figure S14 ***inx-16(ox144)*** **inhibits blue anthranilate and green uranin DF, and loss of punctate staining with uranin and lysotracker.** Worms were killed on agar pads under cover slips with *t*-BOOH.
(PDF)

Figure S15 **Necrosis mutants are not long lived.** (A–F) show lifespan trials with a variety of necrosis mutants. Only *inx-16(ox144)* is longer lived than wild-type (B), and longevity in this slow growing strain may be attributable to dietary restriction. Sample sizes, and probability, *p*, of being the same as N2 control (log rank test): (A) N2, *N* = 114; *vha-2* RNAi, *N* = 98, *p* = 0.67; *vha-12* RNAi, *N* = 103, *p*<0.0001; *cup-5* RNAi, *N* = 104, *p* = 0.031. (B) N2, *N* = 125; *inx-16(ox144)*, *N* = 123, *p* = 0.0047; *cad-1(pj1)*, *N* = 67, *p*<0.0001; *glo-1(zu437)*, *N* = 96, *p* = 0.064; *crt-1(bz29)*, *N* = 110, *p* = 0.34. (C) N2, *N* = 116; *unc-104(e1265)*, *N* = 94, *p* = 0.11; *unc-116(e2310)*, *N* = 89, *p*<0.0001; *glo-1(zu391)*, *N* = 96, *p*<0.05. (D) N2, *N* = 106; *unc-11(e47)*, *N* = 87, *p* = 0.86; *vha-10* RNAi, *N* = 85, *p*<0.0001; *unc-57(e1190)*, *N* = 99, *p*<0.0001; *cad-1(j1)*, *N* = 109, *p* = 0.79. (E) N2, *N* = 110; *vha-10* RNAi, *N* = 87, *p*<0.0001; *cup-5* RNAi, *N* = 102, *p* = 0.82; *asp-3* RNAi, *N* = 104, *p* = 0.71; *asp-4* RNAi, *N* = 97, *p* = 0.63; *clp-1* RNAi, *N* = 103, *p* = 0.86. (F) N2, *N* = 113; *snt-1(md290)*, *N* = 92, *p*<0.0001; *crt-1(ok948)*, *N* = 96, *p* = 0.68. Each panel shows data from a single trial, apart from (B), which shows summed data from two trials.
(PDF)

Figure S16 **Resistance to lethal heat stress (35°C) of necrosis mutants.** There were two independent assays, each with 60 worms (*n* = 120 for all strains). *** *p*<0.001. NS, not significant.
(PDF)

Figure S17 **Effects of anthranilate levels on resistance to lethal stress.** (A–C) Effect on resistance to lethal heat stress (35°C) of manipulation of AA levels using RNAi and AA supplementation. *tdo-2*(RNAi) reduces AA levels (this study, [37]) and increases Trp levels [37]. Restoration of AA in *tdo-2*(RNAi) did not restore stress sensitivity, nor did AA supplementation of wild-type worms increase stress sensitivity. *kmo-1*(RNAi) greatly increases AA levels (this study, [37]) and only marginally increases Trp levels [37]. Probability *p* of being the same as RNAi control calculated using the log rank test. Statistics: (A) RNAi control, *N* = 116, mean survival 15.5 h; *tdo-2*(RNAi), *N* = 111, mean survival 19.9 h, *p*<0.001. (B) RNAi control, *N* = 203, mean survival 11.6 h; *kmo-1*(RNAi), *N* = 178, mean survival 11.1 h, *p* = 0.0037; *tdo-2*(RNAi), *N* = 209, mean survival 12.2 h, *p*<0.001. (C) RNAi control, *N* = 220, mean survival 12.7 h; RNAi control+5 mM AA, *N* = 226, mean survival 12.6 h, *p* = 0.49; *tdo-2*(RNAi), *N* = 254, mean survival 13.3 h, *p*<0.001; *tdo-2*(RNAi)+5 mM AA, *N* = 249, mean survival = 249, *p* = 0.015. (D) Effect of mutations affecting kynurenine pathway on resistance to thermal injury (35°C). Only *flu-2* mutants showed heat stress resistance. Statistics: wild type (N2), *N* = 100, mean survival 11.1 h; *glo-1*, *N* = 100, mean survival 12.3 h, *p*>0.05; *flu-1*, *N* = 100, mean survival 12.3 h, *p*>0.05; *flu-2*, *N* = 100, mean survival >16 h, *p*<0.001; *nkat-1*, *N* = 100, mean survival 12.9 h, *p*>0.05. (E) Effect of mutations affecting kynurenine pathway on resistance to osmotic stress (500 mM NaCl). *glo-1* and *flu-1* decrease AA levels, *flu-1* mutation increases AA, while *nkat-1* has little effect; however, all protect against lethal osmotic stress. Thus, there is no correspondence between AA level and resistance. Statistics: wild type, *N* = 300; *glo-1*, *N* = 250, *p*<0.01; *flu-1*, *N* = 150, *p*<0.01; *flu-2*, *N* = 150, *p*<0.05; *nkat-1*, *N* = 150, *p*<0.05. Probability *p* of being the same as wild type (two-tailed *t* test). * *p*<0.05, ** *p*<0.01. *tdo-2*(RNAi) ±5 mM AA did not affect resistance to osmotic stress (not shown). Taken together, these data imply that AA levels and DF do not affect sensitivity to lethal stress. Effects of *tdo-2*(RNAi) and *flu-2* on heat stress resistance more likely reflects increased Trp levels [37].
(PDF)

Figure S18 **Effect of insulin/IGF-1 signaling on DF.** (A) Death in young adults from lethal stress (freeze-thaw). Three biological replicates of 60 1-d-old adults each (measured in microtitre plates using plate reader). Fluorescence normalized to mean cross-sectional area of intestine. (B) Death in old adults from aging. Sample sizes: N2, 47; *daf-2(e1370)*, 19; *daf-16(mgDf50)*, 20 (measured *in situ* on NGM plates using time lapse photography).
(PDF)

Table S1 **Multiple types of fluorescence increase with death in** ***C. elegans*** **(cf., Figure S5).** Table shows that both green and red fluorescence increase significantly with age, but blue fluorescence does not. Table also shows a significant increase in all types of fluorescence at death, with peak DF significantly higher than at any time seen during life.
(DOCX)

Table S2 **NMR spectroscopic data for angl#1.**
(DOCX)

Table S3 **NMR spectroscopic data for iglu#1.**
(DOCX)

Table S4 **High-resolution MS data angl#1, angl#2, iglu#1, and iglu#2.**
(DOCX)

Video S1 **DF in worms dying from old age (cf.,** **Figure 1** **).** Blue fluorescence in five class C (senescent, non-motile) wild-type worms, viewed with a DAPI filter set, over 5 h. The rise in fluorescence at death coincides with cessation of residual movement.
(MOV)

Video S2 **DF in a young adult worms dying from injury (desiccation) (cf.,** **Figure 2** **).** Blue fluorescence in a young wild-type adult worm dying of desiccation over several hours. Note the rise and spread of DF in an anterior to posterior wave.
(MOV)

Video S3 **DF in a young adult worms dying from injury (heat) (cf.,** **Figure 6** **).** Here death has been induced more rapidly by placing a heated wire immediately adjacent to it. The fluorescence spreads down the length of the animal over the 6 min of filming.
(MOV)

Video S4 **DF does not spread in an** ***inx-16*** **mutant (cf.,** **Figure 6** **).** Here a young adult *inx-16* mutant adult on an agar plate is killed by placing a hot platinum wire (worm pick) immediately adjacent to it. The fluorescence does not spread down the length of the animal and only fades in intensity during the 6 min of filming.
(MOV)

Video S5 **Spread of a wave of calcium influx during death (cf.,** **Figure 6** **).** Here in a dying worm calcium levels rise in the anterior intestine. High levels of calcium appear red. The anterior intestine is at the top.
(AVI)

Video S6 **Waves of cytosolic acidosis in the intestine during death (cf.,** **Figure 6** **).** Here a worm killed using *tert*-butylhydroperoxide exhibits a wave of cytosolic acidosis in the intestine. The anterior intestine is at the top right. Higher pH (∼7.4), red, changing via green to lower pH, dark blue (∼6.4) (see Figure S13D for full pH color key).
(WMV)

## Abbreviations

AA: anthranilic acid
DANS: differential analysis by 2D-NMR spectroscopy
DF: death fluorescence
ER: endoplasmic reticulum
LMP: lysosomal membrane permeabilization
NGM: nematode growth medium
