# Supplementary figures and images for: Anthranilate Fluorescence Marks a Calcium-Propagated Necrotic Wave That Promotes Organismal Death in C. elegans
Source: PLoS Biol. 2013 Jul 23;11(7):e1001613. doi: 10.1371/journal.pbio.1001613 (PMC3720247; doi:10.1371/journal.pbio.1001613)

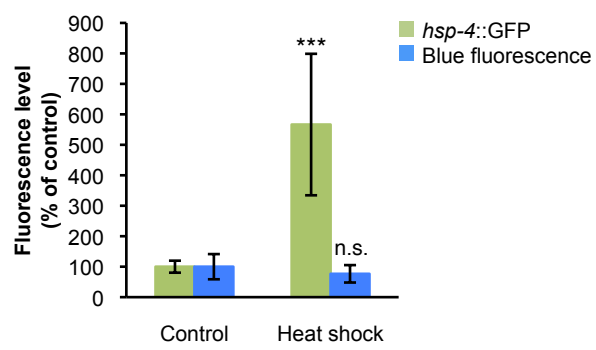

Supplement: Figure S1 — Blue fluorescence levels do not increase with increased expression of an unfolded protein response (UPR) associated gene. After exposure to elevated heat levels (30°C, 3 h), hsp-4::GFP levels rise significantly but blue fluorescence levels remain unaffected. Mean of three biological replicates, ± SD, *** p<0.001, Student's t test. (PDF) [file pbio.1001613.s001.pdf]

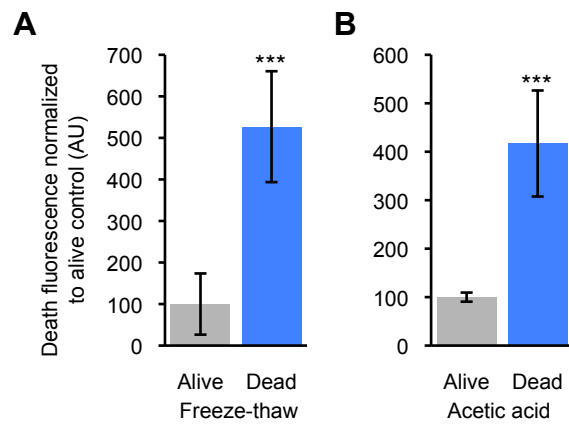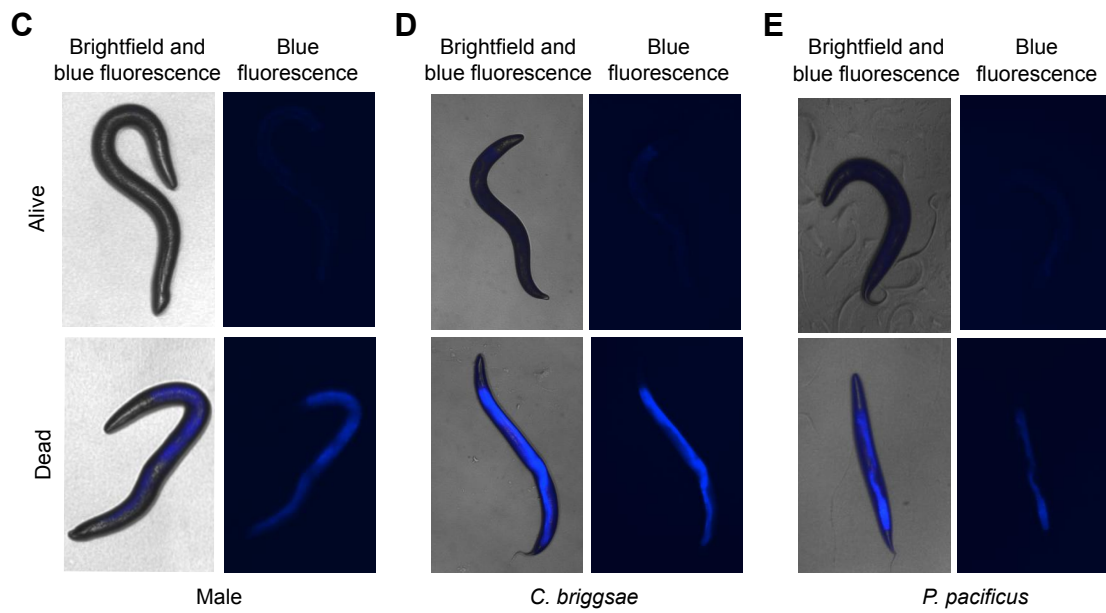

Supplement: Figure S2 — DF is induced by different methods of killing. (A) DF when C. elegans are killed by freeze-thaw. Mean of 3 biological replicates, 60 worms per trial, ± SD, *** p<0.001. (B) DF when C. elegans are killed by low pH (acetic acid, pH 3). Mean of 3 replicates, 30 worms per trial. (C) Representative images of DF in young adult wild-type males. (D, E) DF in other nematode species. Representative images of DF in C. briggsae and P. pacificus. (PDF) [file pbio.1001613.s002.pdf]

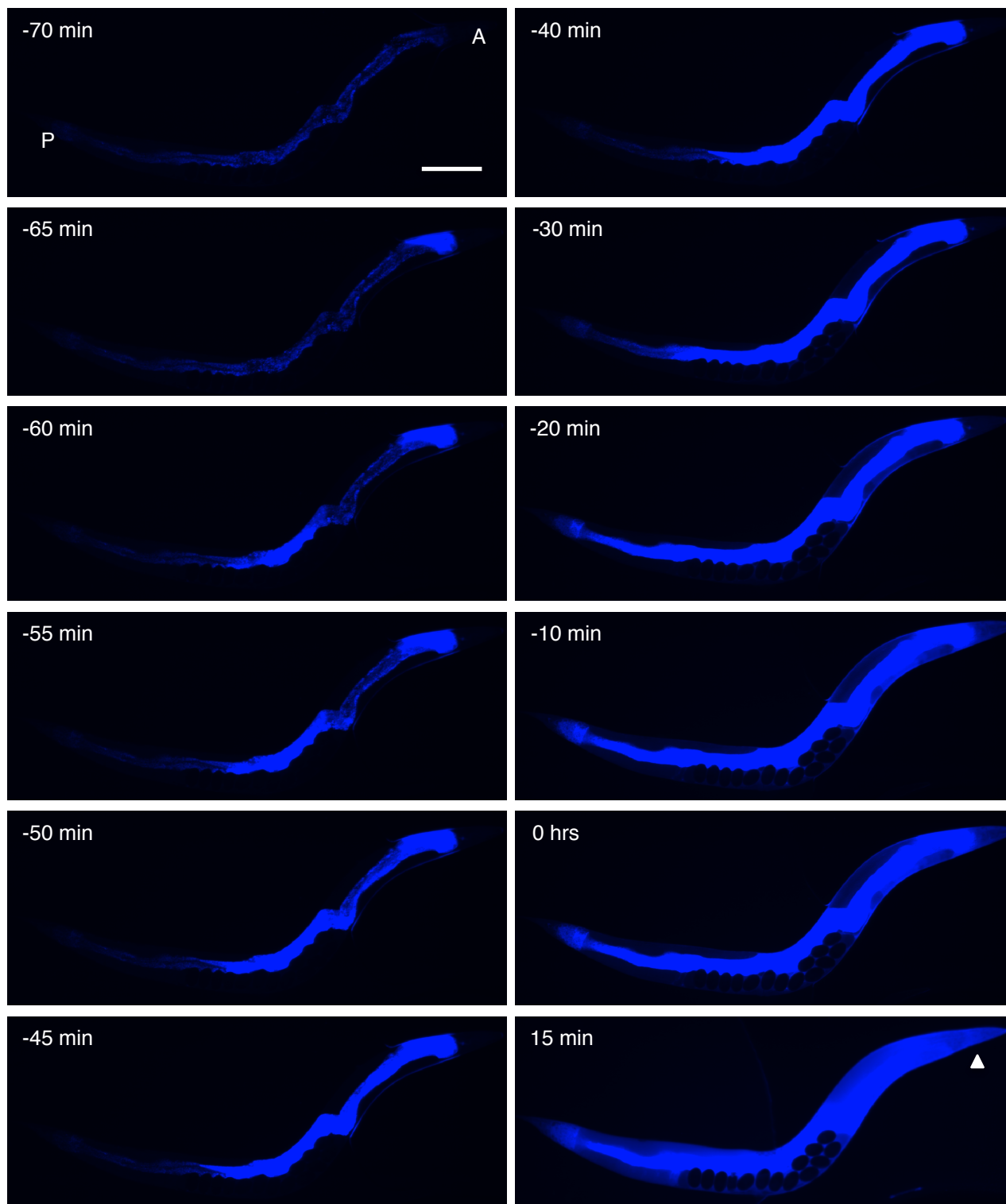

Supplement: Figure S3 — DF in young adult hermaphrodite killed with a heated wire. During DF the pattern of fluorescence changes from punctate to diffuse. Note the secondary focus of DF that appears in the mid-body region; these occur in a proportion of animals. A and P, anterior and posterior ends of intestine. Arrowheads, spread of DF from intestine to other tissues. Scale bar, 200 µm. (PDF) [file pbio.1001613.s003.pdf]

**A**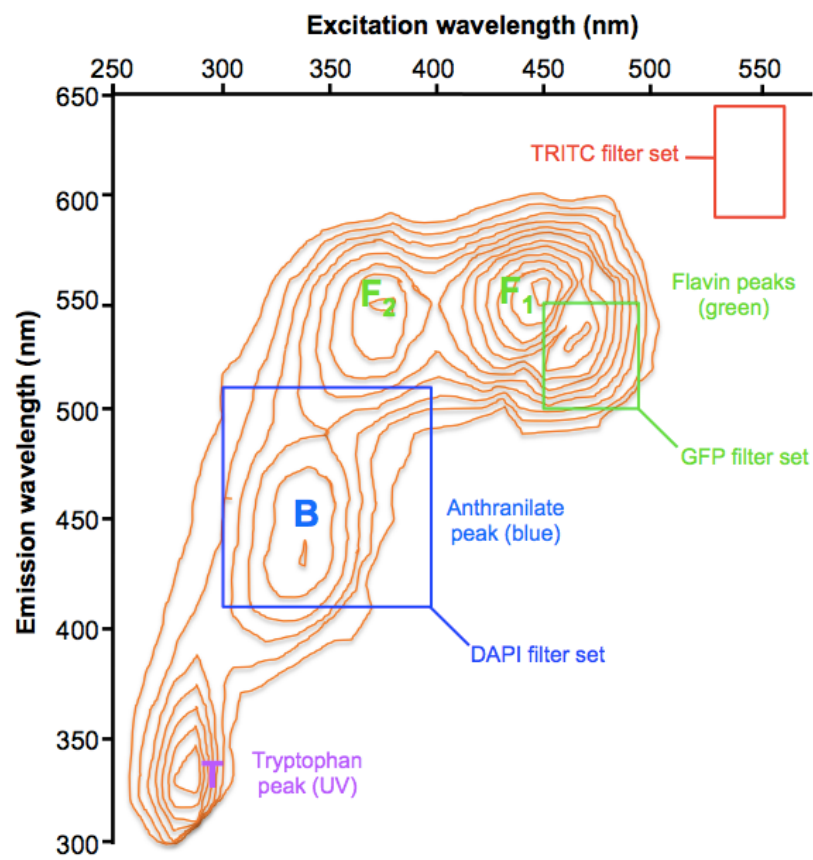**B**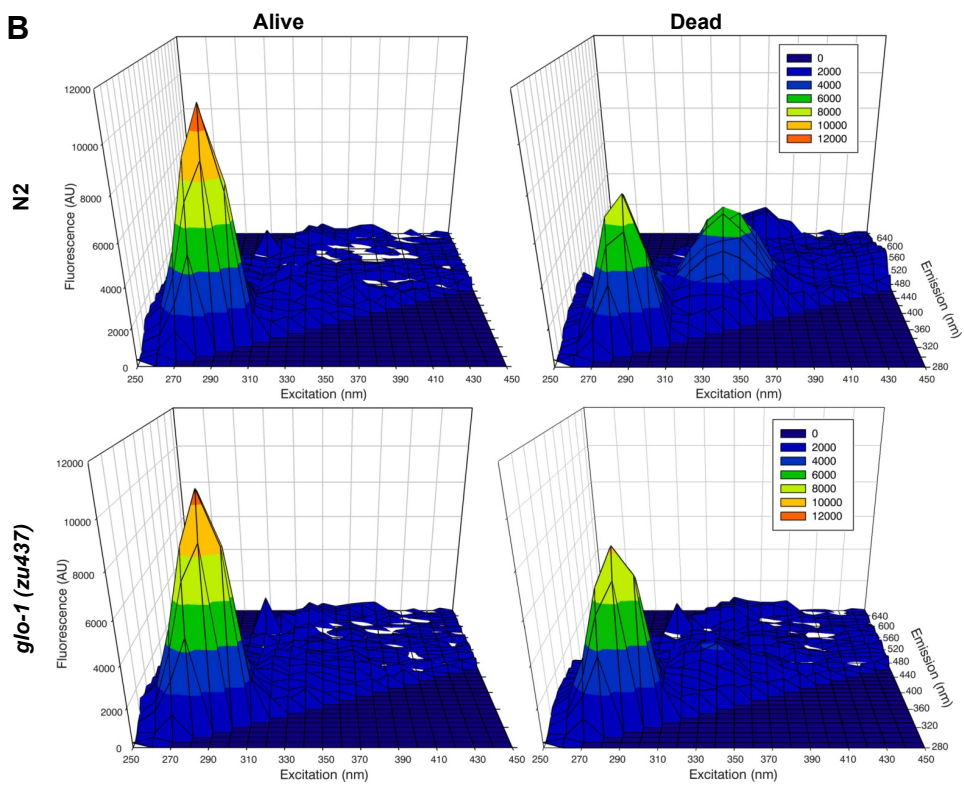

Supplement: Figure S4 — Relationship between major forms of C. elegans fluorescence and filter sets used in this study. This scheme shows a typical full excitation/emission fluorescence spectrum (aqueous homogenate of 12-d-old worms, redrawn from [21]), and their relationship to the specifications of the filter sets used here. B, blue peak, arising from anthranilic acid glucosyl esters. T, ultraviolet peak attributed to tryptophan. F1 and F2, green fluorescence peaks attributed to flavins [21]. (B) At death (freeze-thaw), a large DF peak at λex/λem 340/430 nm appears in wild type (N2), but not in glo-1 animals, which lack gut granules. White patches are negative values. (PDF) [file pbio.1001613.s004.pdf]

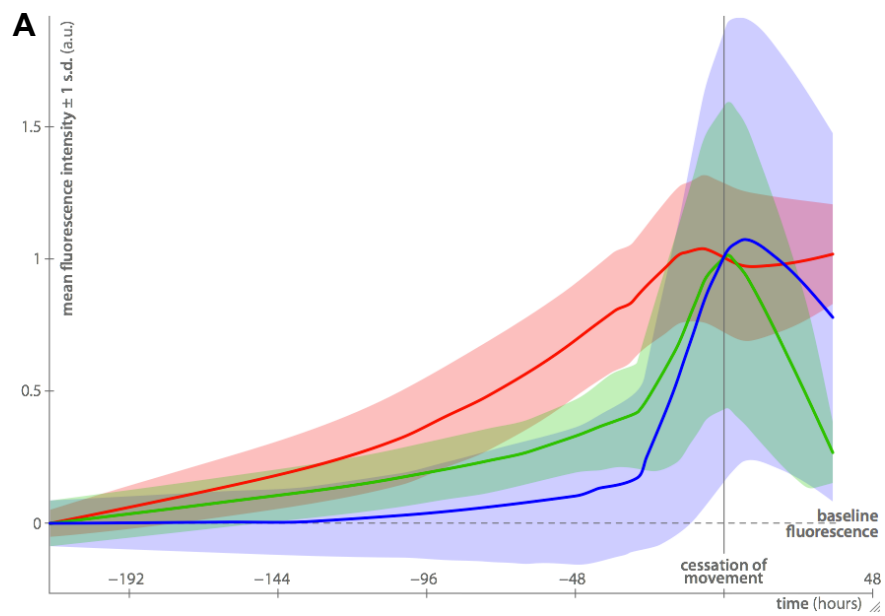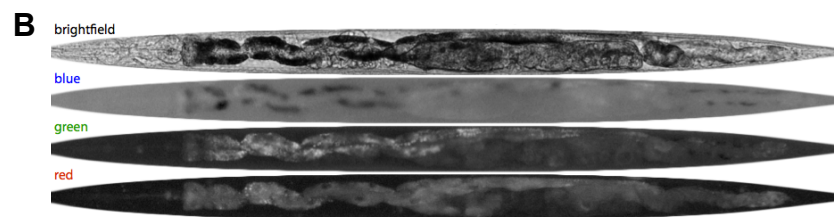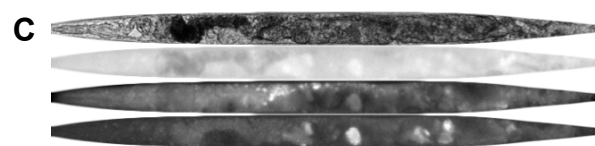

Supplement: Figure S5 — Other sources of fluorescence increase at death in C. elegans . (A) Average levels of fluorescence normalized to time of death, viewed under red, blue, and green filter sets (λex/λem, respectively, TRITC 546/600 nm, DAPI 350/460 nm, and GFP 470/525 nm). Different types of fluorescence showed different rates of increase with time. Forty-three individual animals were measured under each filter set. Shaded area, ±1 SD. Each plot is scaled on separate arbitrary units as absolute-terms comparisons are not possible. Note that rates of fluorescence accumulation and spatial distribution of the blue, red, and green fluorescence differ, consistent with the presence of distinct fluorophores. (B and C) Typical images of worms prior to death (B) and during death (C) in each fluorescence band, showing altered spatial distribution of the fluorescence. (PDF) [file pbio.1001613.s005.pdf]

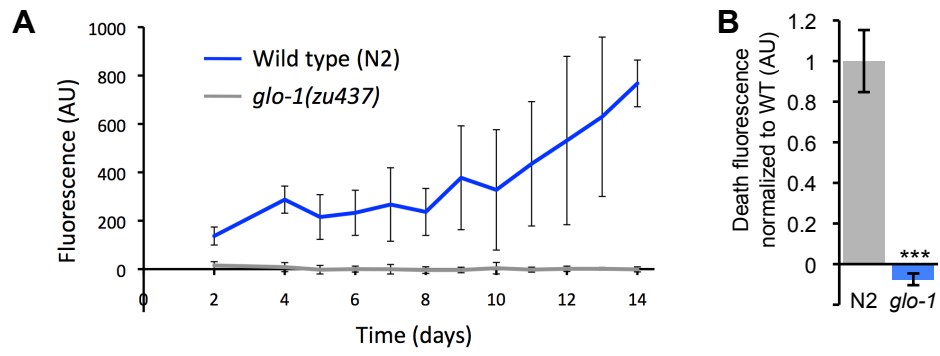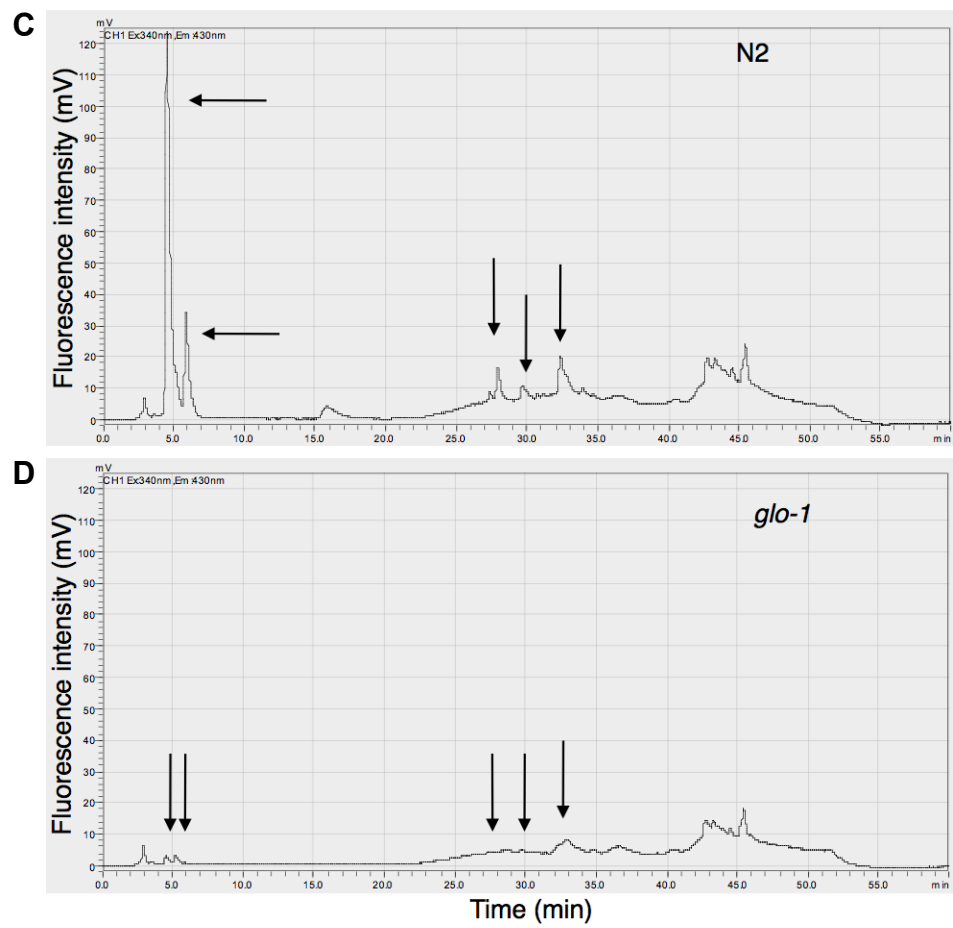

Supplement: Figure S6 — glo-1 animals do not show gut granule or DF. (A and B) glo-1(zu347) animals do not show an increase in blue fluorescence during aging (A) nor DF upon killing (B), making them a useful negative control for establishing the chemical source of gut granule fluorescence and DF. Mean ± SD. Note that the gradual increase in mean blue fluorescence in wild-type worms in (A) reflects an age increase in the proportion of dying worms. (C and D) HPLC chromatogram reveals four peaks present in N2 but not glo-1 animals, marked by black arrows. (PDF) [file pbio.1001613.s006.pdf]

A

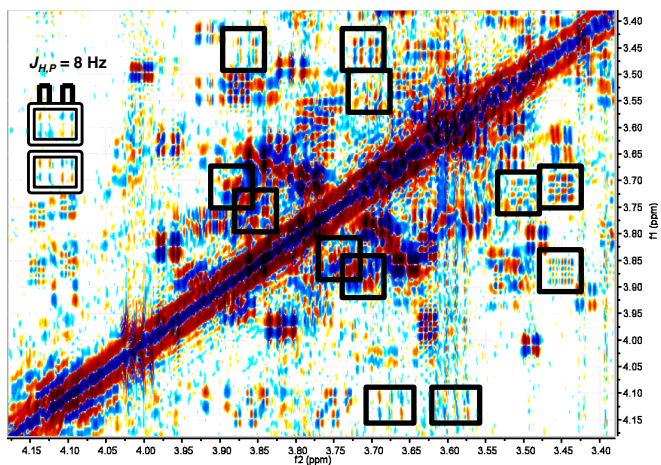

B

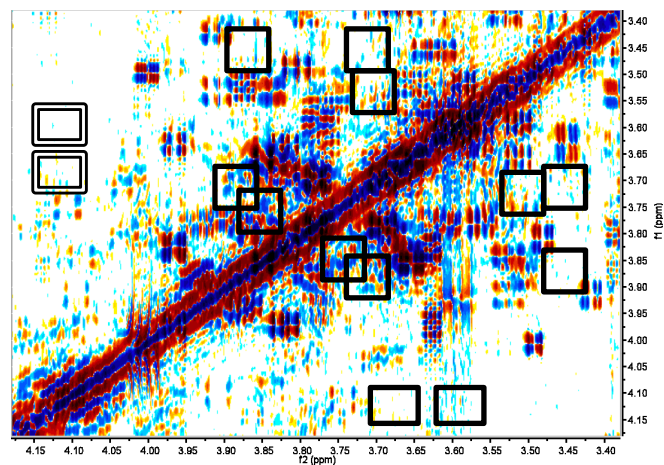

C

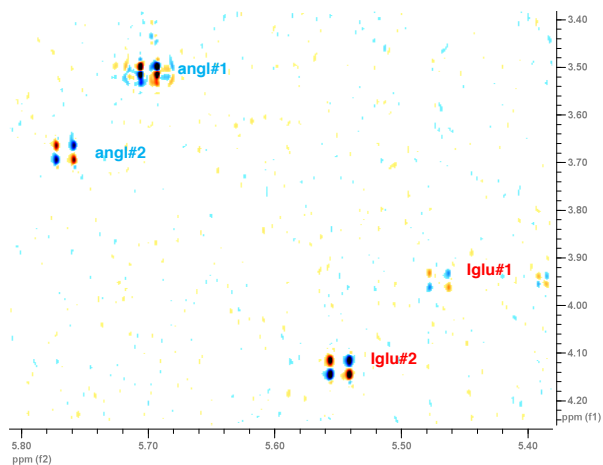

D

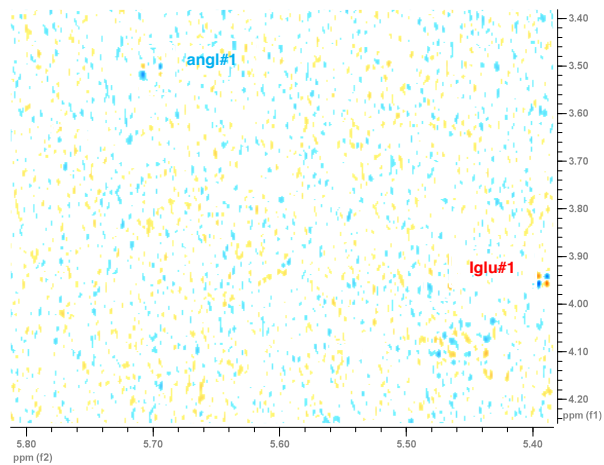

Supplement: Figure S7 — Biochemical identification of DF constituents. (A) 3.4–4.2 ppm region of the dqfCOSY spectrum (600 MHz, methanol-d4) of N2 worm extracts show cross-peaks representing several glucose moieties (black boxes). Crosspeaks at f2 = 4.12 ppm correspond to proton 3-H in angl#2 and show additional J-splitting due to coupling with 31P of the adjacent phosphate group (double bordered boxes). (B) 3.4–4.2 ppm region of the dqfCOSY spectrum (600 MHz, methanol-d4) of glo-1(zu437) worm extracts do not show cross-peaks representative of the glucose moieties present in N2 worm extracts (black boxes). (C) Section of the dqfCOSY spectrum (600 MHz, methanol-d4) of N2 worm extracts showing crosspeaks for the anomeric protons of four glucose units. Two of these (red boxes) belong to the indole glucosides (iglu#1, iglu#2) and the other two (blue boxes) are part of the anthranilic acid glucosides (angl#1, angl#2). (D) Section of the dqfCOSY spectrum (600 MHz, methanol-d4) of glo-1 worm extracts corresponding to the section of the N2 dqfCOSY spectrum shown in (C). Cross-peaks representing indole glucosides (iglu#1, iglu#2) and anthranilic acid glucosides (angl#1, angl#2) are much weaker or completely absent. (PDF) [file pbio.1001613.s007.pdf]

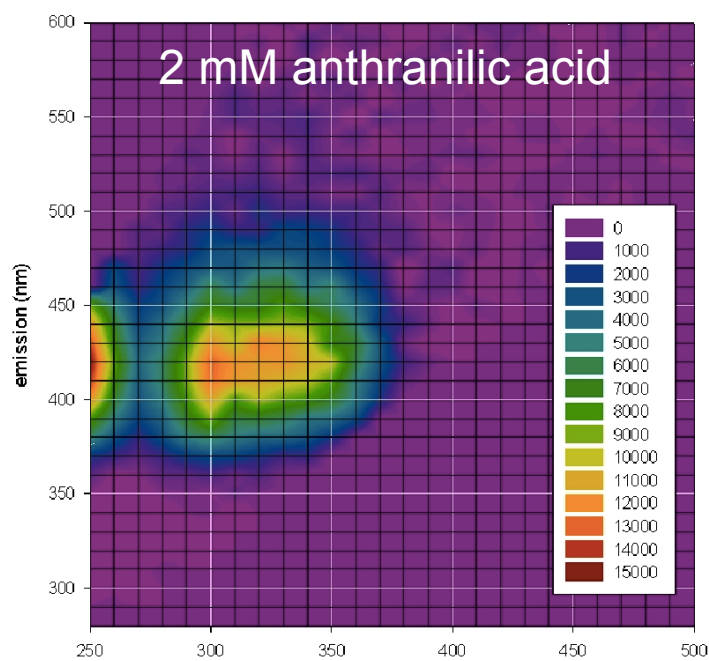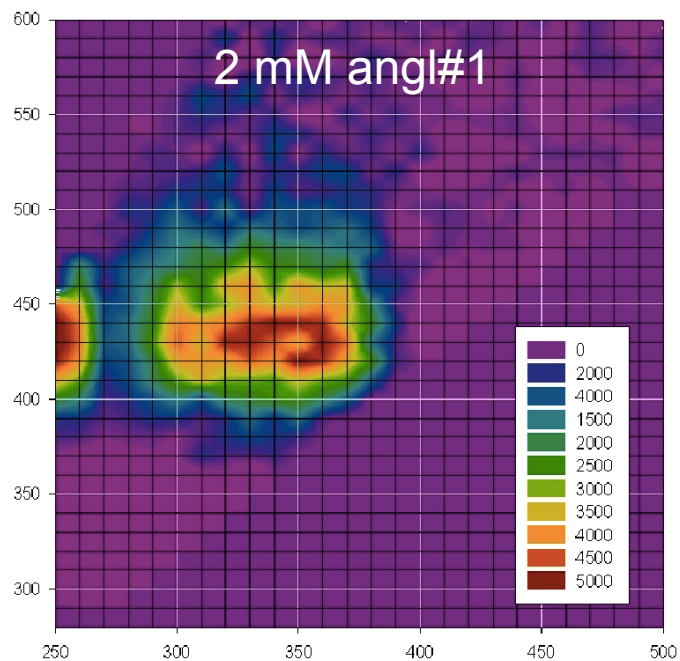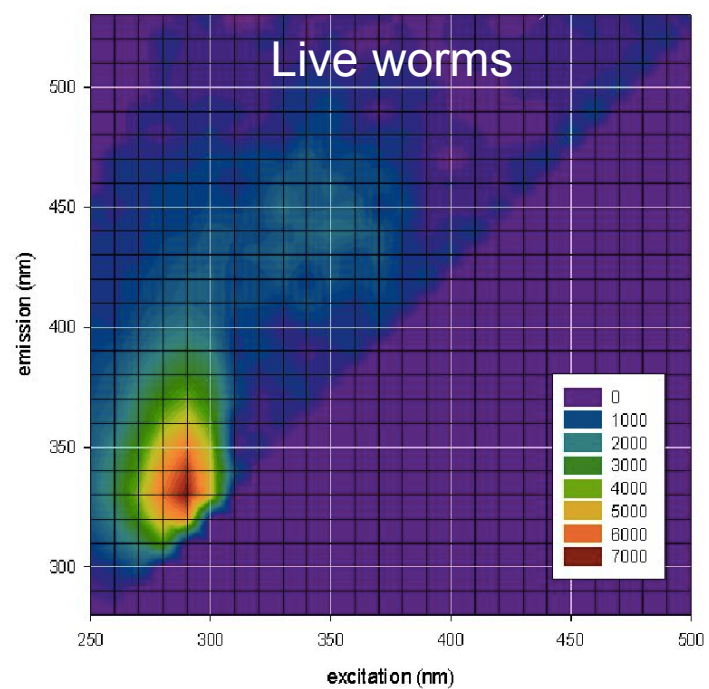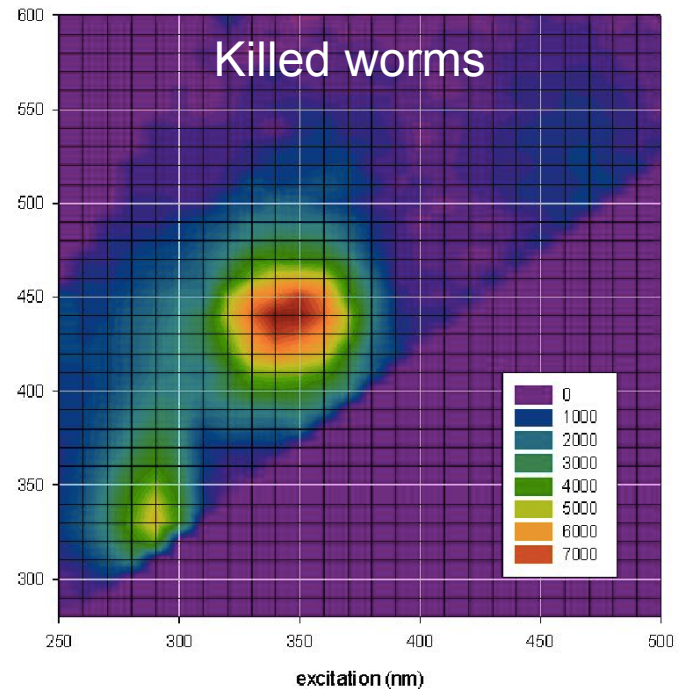

Supplement: Figure S8 — Fluorescence spectra for angl#1 and worm blue fluorescence are highly similar. Also shown is fluorescence of unconjugated anthranilic acid at the same concentration as angl#1 (2 mM). Note that AA fluorescence is less similar to worm blue fluorescence than that of angl#1. The blue fluorescence at λex<270 nM in angl#1 and AA may be suppressed in the worm by the presence of other substances; we noted that addition of worm lysate to AA markedly reduced this fluorescence (unpublished data). (PDF) [file pbio.1001613.s008.pdf]

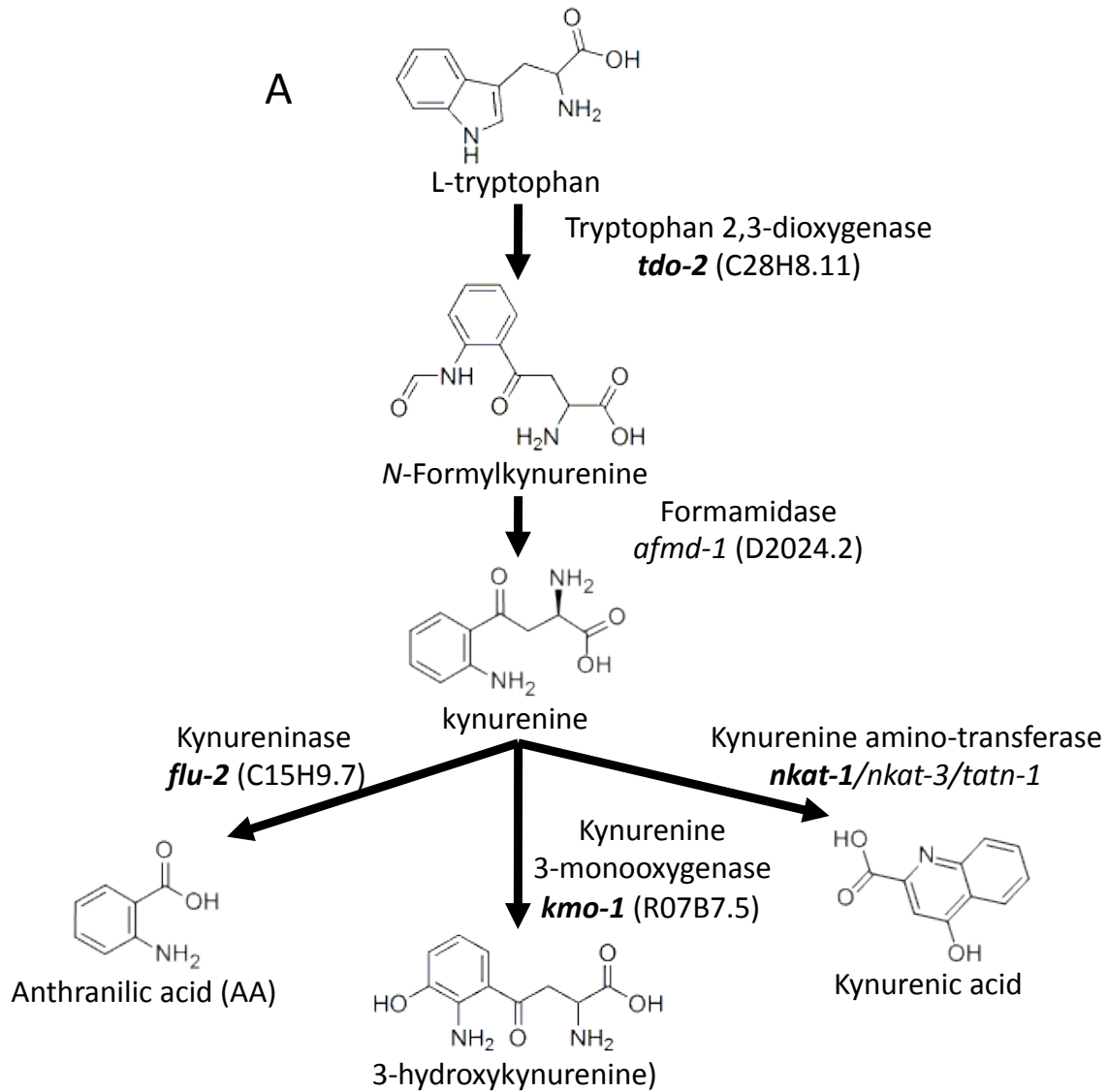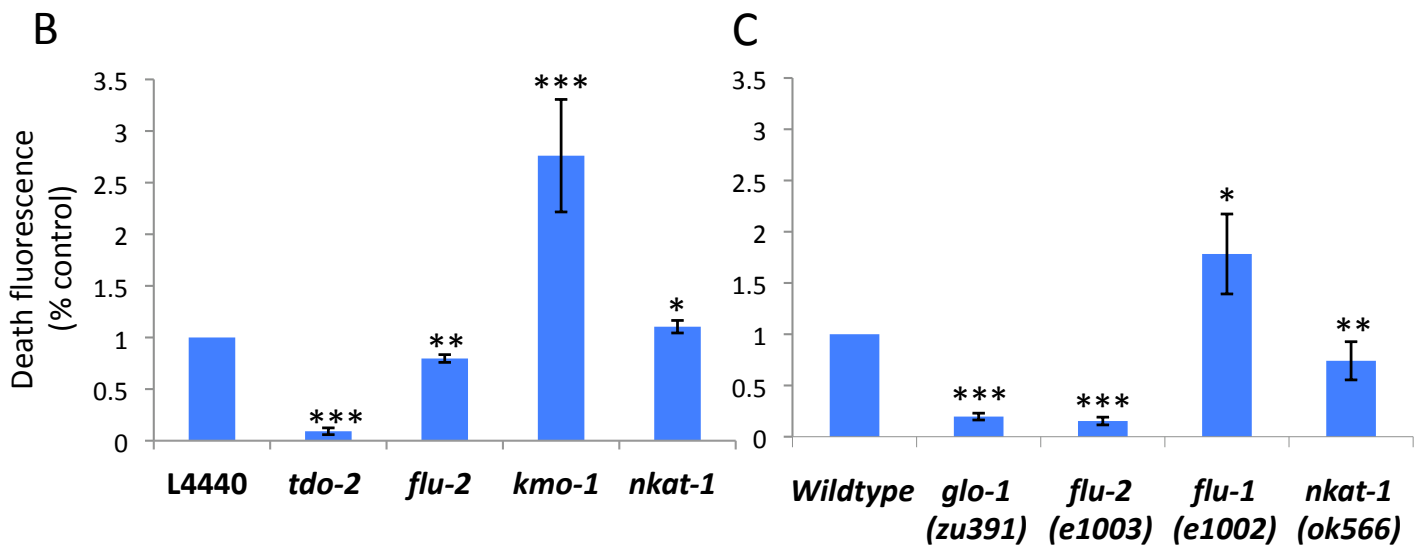

Supplement: Figure S9 — Kynurenine pathway genes affect DF levels. (A) The kynurenine pathway (upper portion). (B) Effects of RNAi of kynurenine pathway genes on DF. Worms were killed using freeze-thaw, and fluorescence measured in a plate reader. tdo-2 and flu-2 promote AA production, and here RNAi reduced DF, as expected. kmo-1 and nkat-1 convert kynurenine into compounds other than AA. Thus, kmo-1(RNAi) increased AA levels, likely by increasing kynurenine availability for AA synthesis. By contrast, nkat-1(RNAi) only marginally increased AA levels. This suggests that in wild type there is substantial conversion of kynurenine to 3-hydroxykynurenine but not kynurenic acid. The effect of nkat-2(ok566) (see C) is consistent with this. (C) Effects of mutation of kynurenine pathway genes on DF. flu-2(e1003) greatly reduced DF, to levels similar to those in glo-1(zu437) mutants that lack gut granules. The weaker effect on DF of flu-2(RNAi) in (B) may reflect incomplete abrogation of flu-2 expression. It is likely that flu-1 V and kmo-1 V are the same gene, since mutation of flu-1 greatly reduced activity levels of kynurenine 3-monooxygenase (kynurenine hydroxylase) [73]. Consistent with this, flu-1(e1002), like kmo-1(RNAi), greatly increased AA levels. (PDF) [file pbio.1001613.s009.pdf]

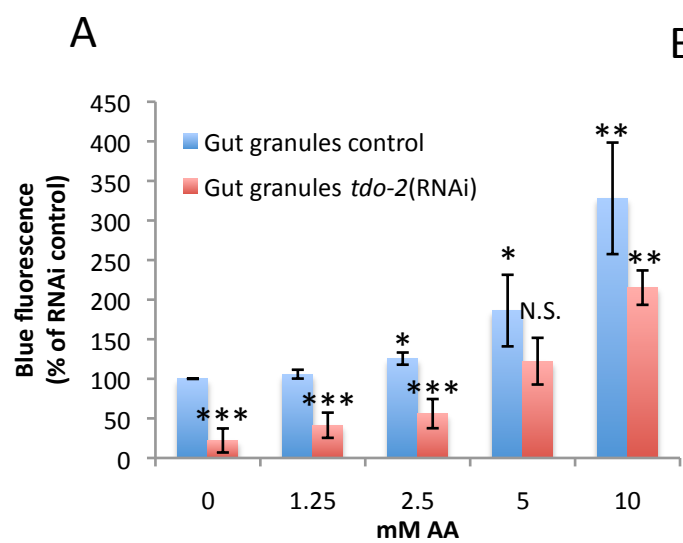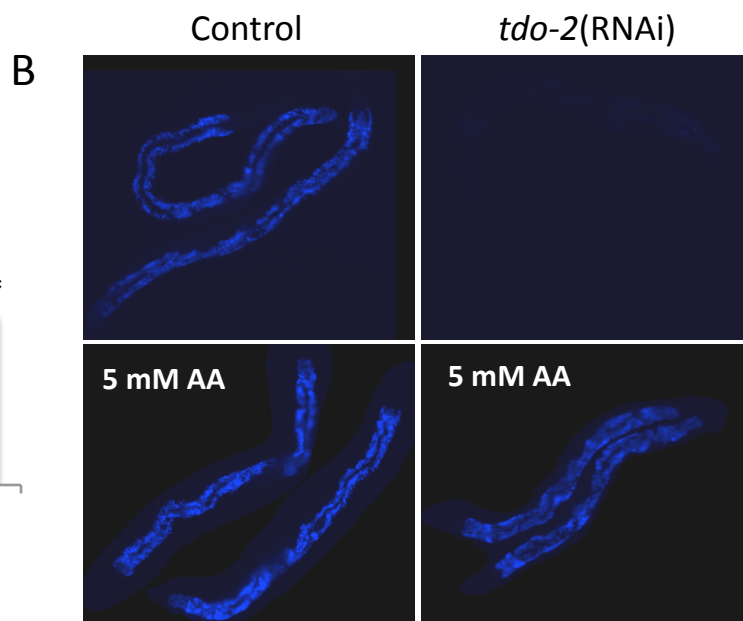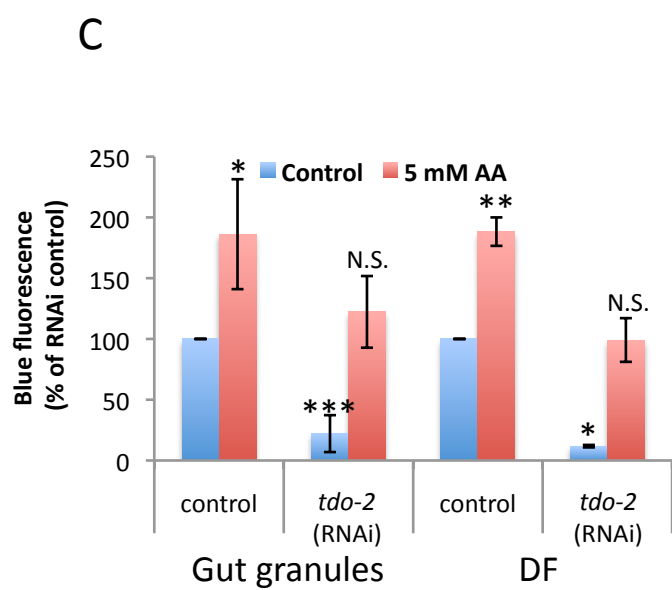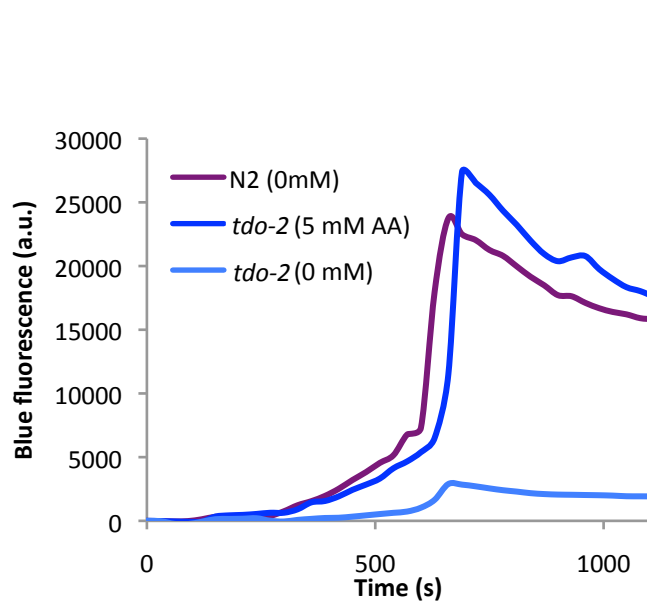

Supplement: Figure S10 — Anthranilic acid (AA) supplementation rescues gut granule and DF. (A, B) Incubation of tdo-2(RNAi) worms in 5 mM AA leads to gut granule fluorescence similar to wild type. (A) Effect on gut granule fluorescence of incubation in a range of AA concentrations. 5 mM AA gives fluorescence levels similar to wild type. (B) Epifluorescence microscopy reveals restoration of gut granule fluorescence by incubation in 5 mM AA. (C, D) Incubation of tdo-2(RNAi) worms in 5 mM AA leads to DF similar to wild type. (C) Effects of tdo-2(RNAi) and AA supplementation on peak DF. (D) Similar kinetics of DF in control worms and tdo-2(RNAi) worms with AA-replenished gut granules, even though only the former can synthesize AA. (PDF) [file pbio.1001613.s010.pdf]

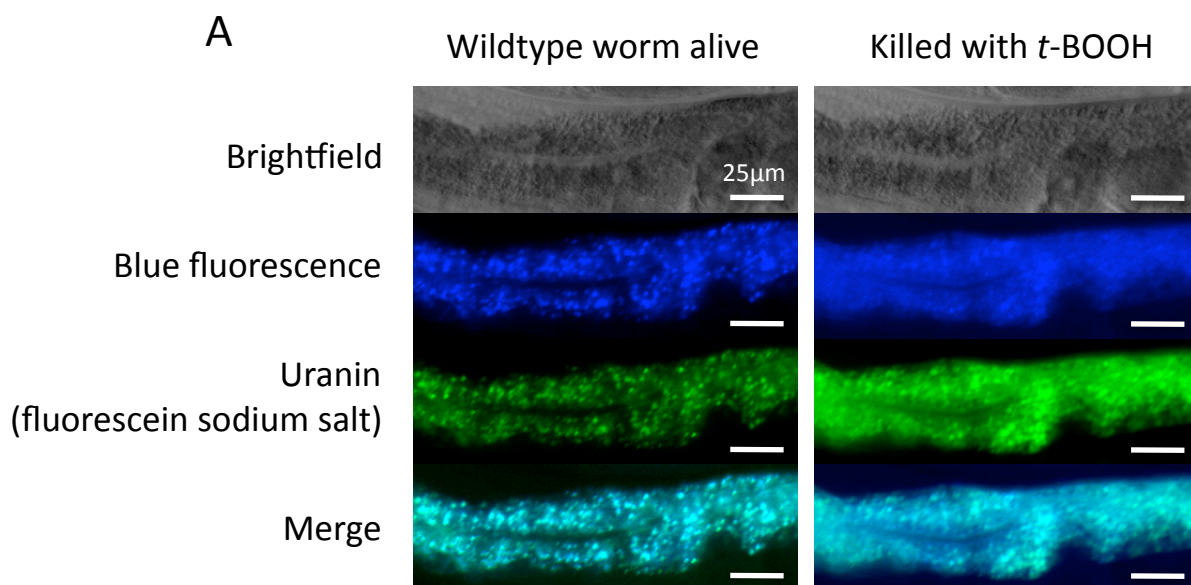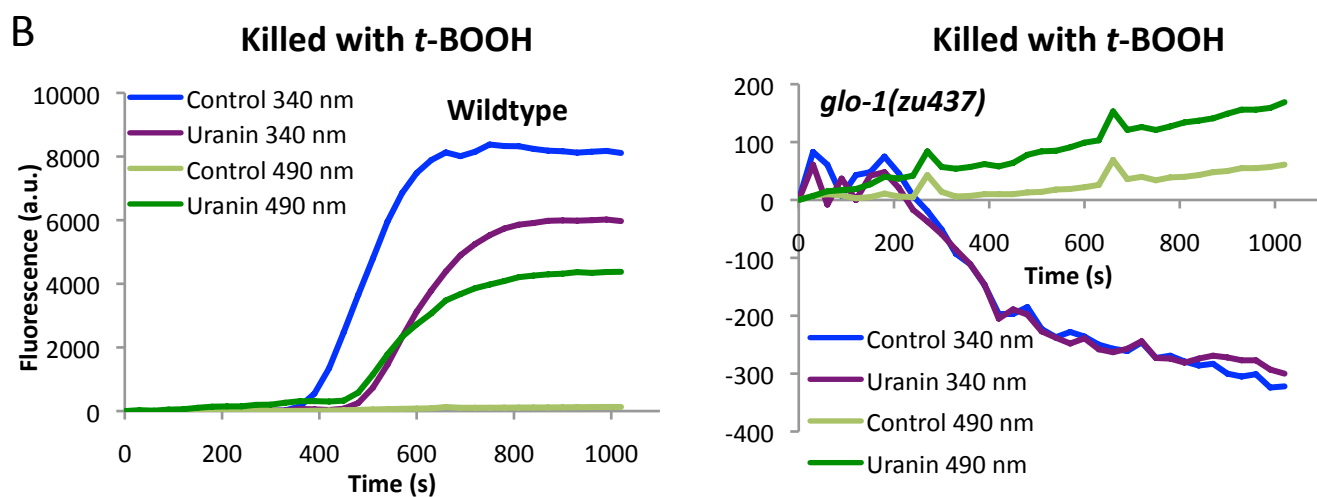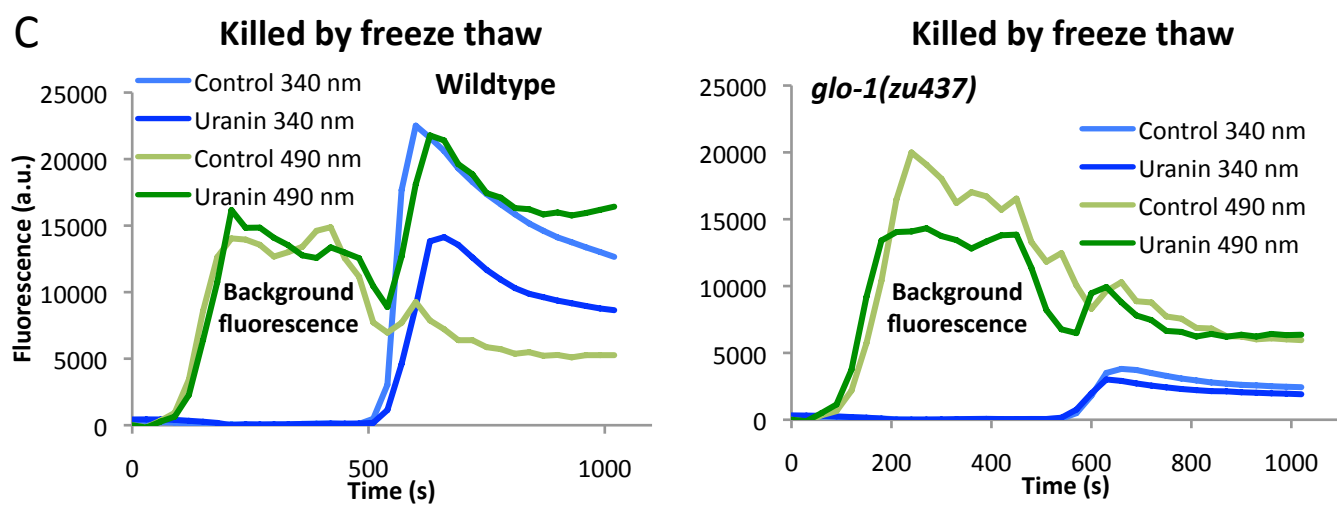

Supplement: Figure S11 — Uranin treatment leads to green DF. (A) Treatment with uranin leads to punctate green fluorescence that co-localizes with blue gut granule fluorescence. (B and C) Worms were incubated with uranin and then killed. (B) Killing with t-BOOH and (C) with freeze-thaw. Killing of uranin-treated worms led to a burst of green fluorescence in the wild type (N2) but not in a glo-1(zu437) mutant, which has no gut granules. Anthranilate and uranin fluorescence do not overlap: in the absence of uranin, there was no increase in λem 490 nm fluorescence at death, nor did the presence of uranin increase λem 340 nm fluorescence at death. For unknown reasons, uranin caused a slight decrease and delay in λem 340 nm fluorescence at death, in both forms of killing. Note that freeze-thaw killing caused substantial background λem 490 nm fluorescence. (PDF) [file pbio.1001613.s011.pdf]

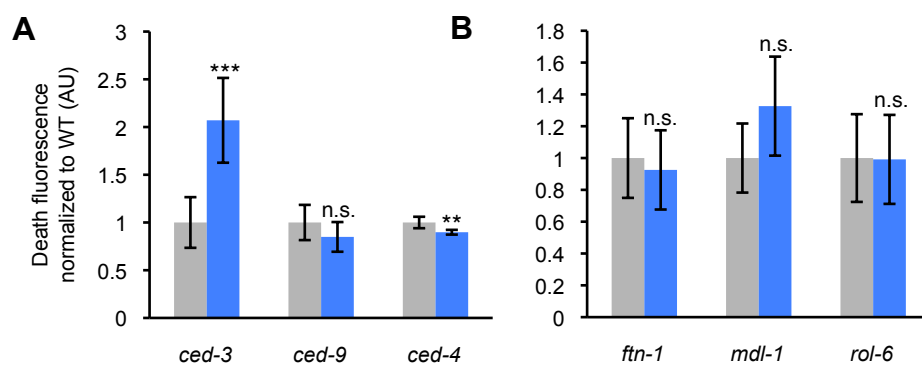

Supplement: Figure S12 — Mutants not predicted to have reduced necrosis do not show decreased DF. These are negative controls for data shown in Figure 5. Graphs show relative increase of fluorescence at death, induced by freeze-thaw (60 young adult worms/strain/replicate). Mean ± SD, 3 biological replicates, **p<0.01 and ***p<0.001. (A) Apoptosis defective mutants. These results imply that apoptosis does not contribute to DF. ced-3 mutants show significantly increased DF, perhaps reflecting inhibition of necrosis by wild-type ced-3. (B) Random selection of mutants unconnected to cell death, which do not show any significant change in DF. The mutations were in the genes ftn-1, mdl-1, and rol-6 (encoding ferritin, the MAD-like transcription factor and a cuticular collagen, respectively). Selection of these mutants as negative controls was on the basis of lack of a known association with necrosis, and availability in the laboratory. (PDF) [file pbio.1001613.s012.pdf]

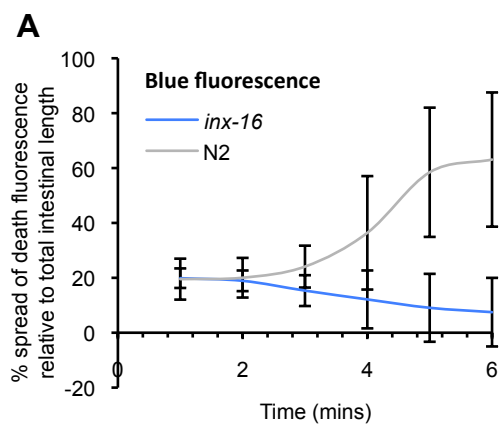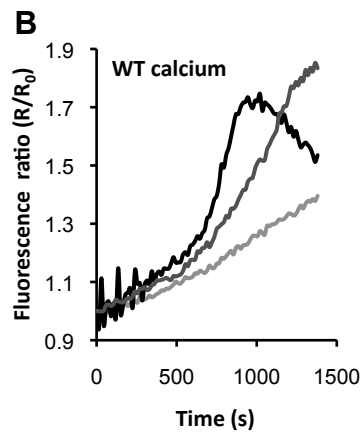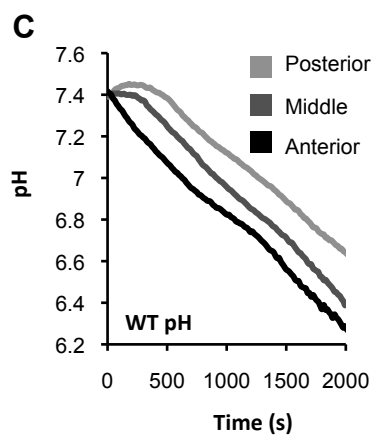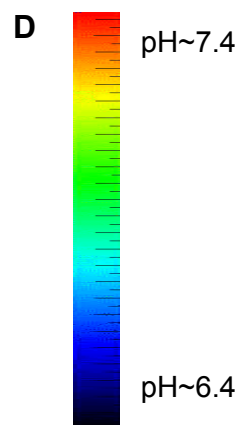

Supplement: Figure S13 — Calcium spread and acidosis are correlated with DF. (A) Loss of inx-16 prevents the spread of DF. (B and C) Ca2+ levels and pH in the intestine of worms killed by oxidative stress (t-BOOH). (B) In vivo Ca2+ levels rise at death in the anterior intestine prior to the posterior intestine, consistent with an anterior to posterior Ca2+ wave. (C) In vivo intestinal pH decreases at death from pH ∼7.4 to ∼6.3, and this occurs more rapidly in the anterior than the posterior intestine, consistent with an anterior to posterior wave of cytosolic acidosis. Mean ± SD. Here “anterior” indicates the int1 and int2 anterior intestinal cells, “middle” the juxta-vulva region (∼int5), and “posterior” the int9 posterior intestinal cells. (D) Key to pH sensor in Video S6. (PDF) [file pbio.1001613.s013.pdf]

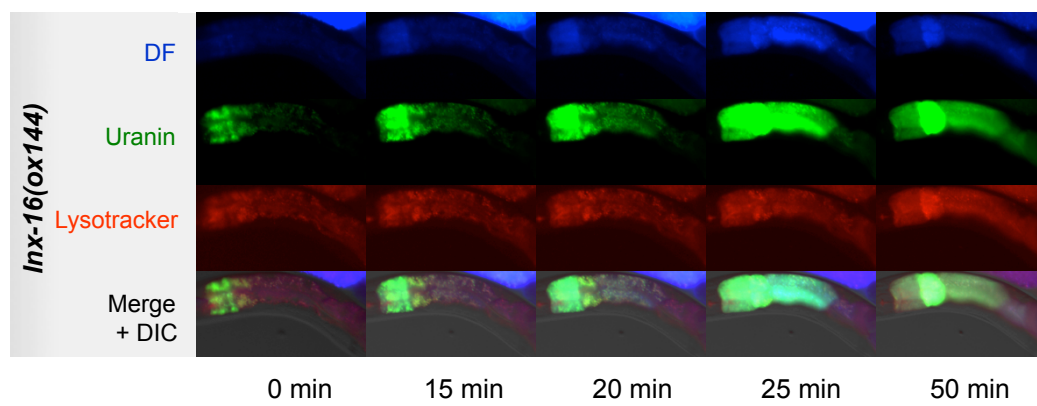

Supplement: Figure S14 — inx-16(ox144) inhibits blue anthranilate and green uranin DF, and loss of punctate staining with uranin and lysotracker. Worms were killed on agar pads under cover slips with t-BOOH. (PDF) [file pbio.1001613.s014.pdf]

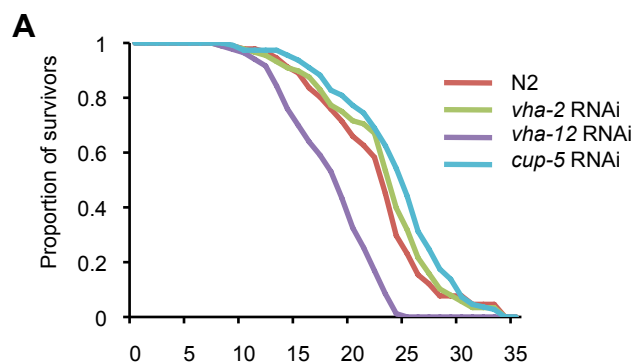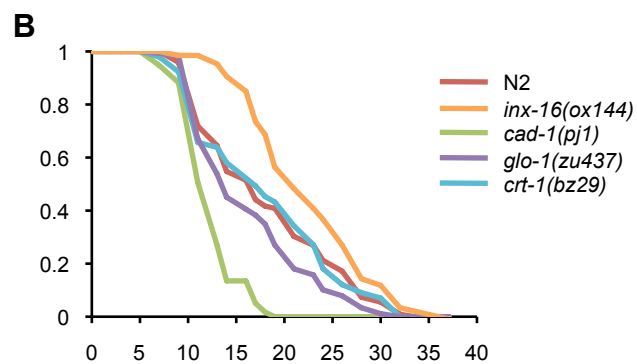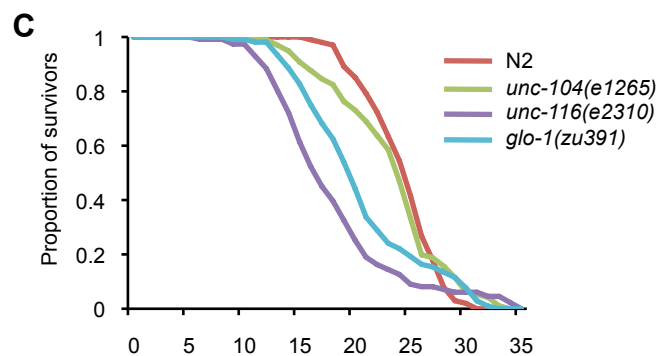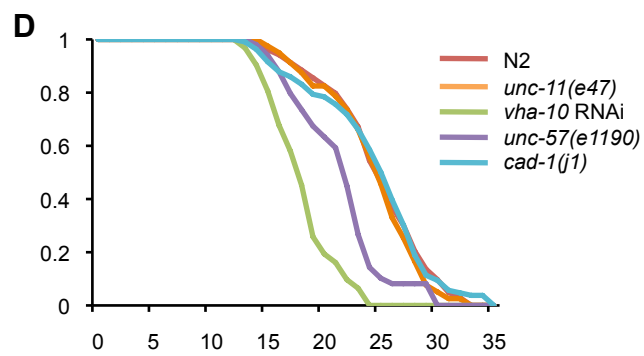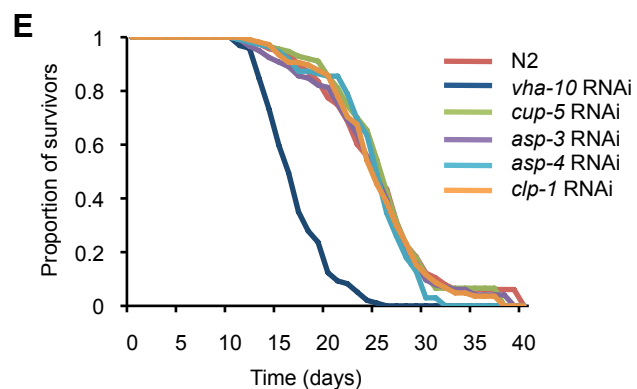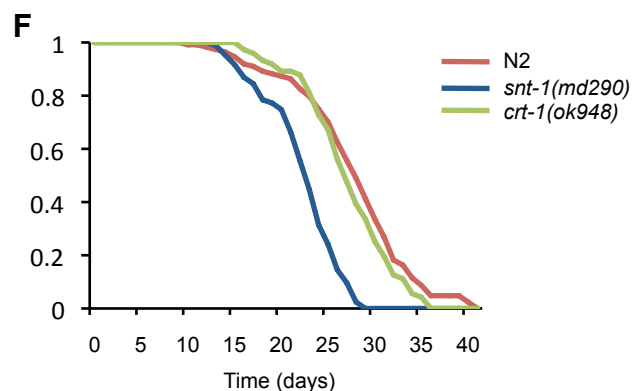

Supplement: Figure S15 — Necrosis mutants are not long lived. (A–F) show lifespan trials with a variety of necrosis mutants. Only inx-16(ox144) is longer lived than wild-type (B), and longevity in this slow growing strain may be attributable to dietary restriction. Sample sizes, and probability, p, of being the same as N2 control (log rank test): (A) N2, N = 114; vha-2 RNAi, N = 98, p = 0.67; vha-12 RNAi, N = 103, p<0.0001; cup-5 RNAi, N = 104, p = 0.031. (B) N2, N = 125; inx-16(ox144), N = 123, p = 0.0047; cad-1(pj1), N = 67, p<0.0001; glo-1(zu437), N = 96, p = 0.064; crt-1(bz29), N = 110, p = 0.34. (C) N2, N = 116; unc-104(e1265), N = 94, p = 0.11; unc-116(e2310), N = 89, p<0.0001; glo-1(zu391), N = 96, p<0.05. (D) N2, N = 106; unc-11(e47), N = 87, p = 0.86; vha-10 RNAi, N = 85, p<0.0001; unc-57(e1190), N = 99, p<0.0001; cad-1(j1), N = 109, p = 0.79. (E) N2, N = 110; vha-10 RNAi, N = 87, p<0.0001; cup-5 RNAi, N = 102, p = 0.82; asp-3 RNAi, N = 104, p = 0.71; asp-4 RNAi, N = 97, p = 0.63; clp-1 RNAi, N = 103, p = 0.86. (F) N2, N = 113; snt-1(md290), N = 92, p<0.0001; crt-1(ok948), N = 96, p = 0.68. Each panel shows data from a single trial, apart from (B), which shows summed data from two trials. (PDF) [file pbio.1001613.s015.pdf]

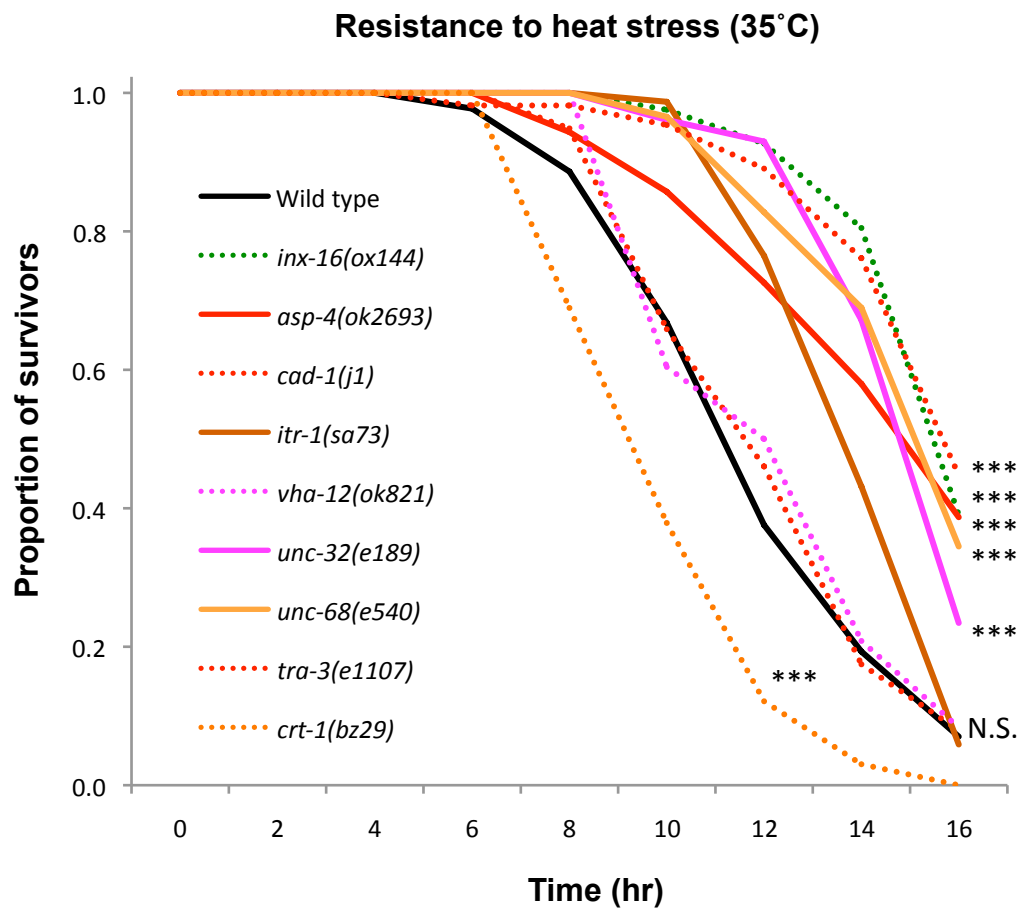

Supplement: Figure S16 — Resistance to lethal heat stress (35°C) of necrosis mutants. There were two independent assays, each with 60 worms (n = 120 for all strains). *** p<0.001. NS, not significant. (PDF) [file pbio.1001613.s016.pdf]

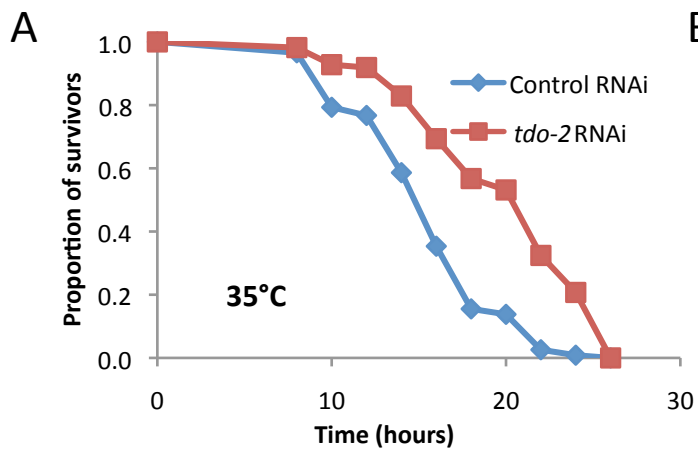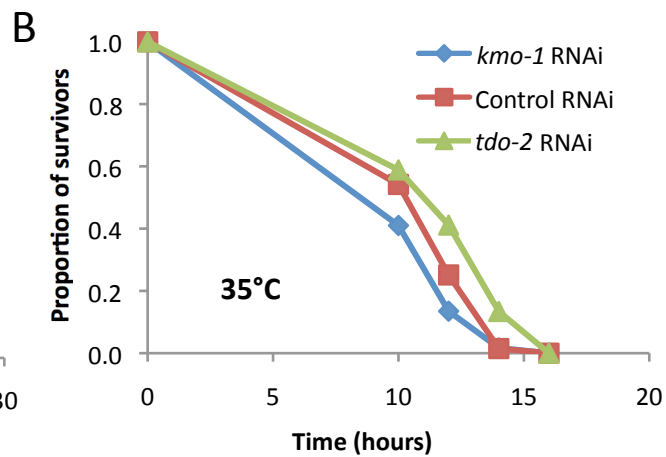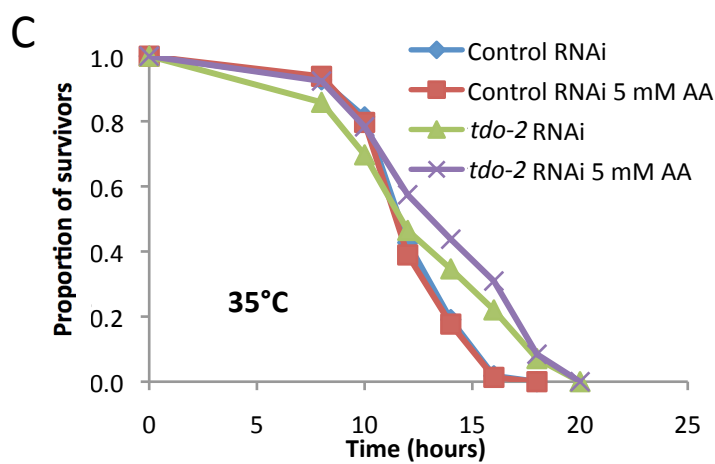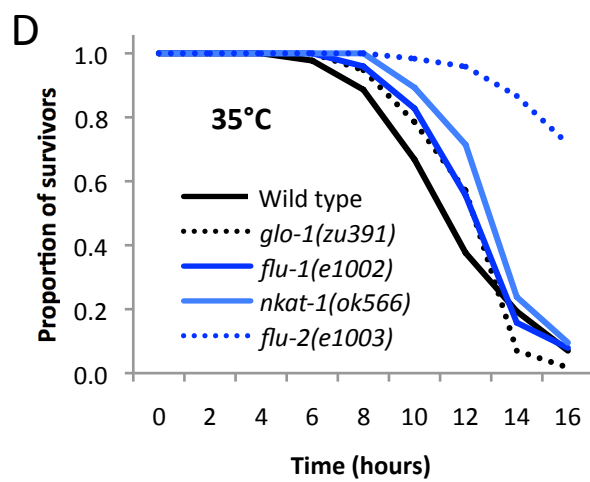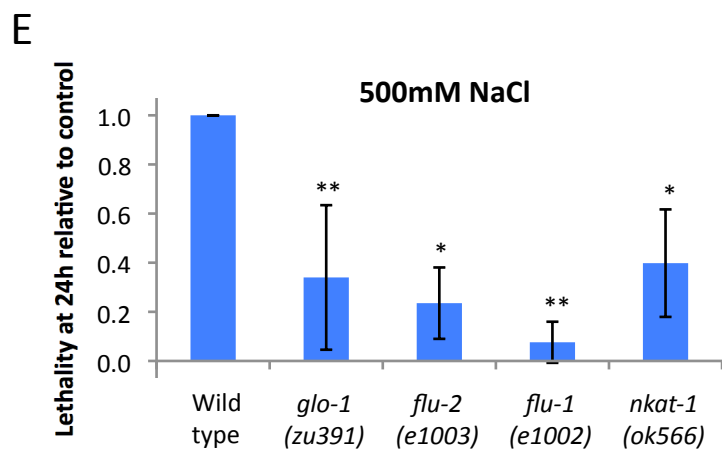

Supplement: Figure S17 — Effects of anthranilate levels on resistance to lethal stress. (A–C) Effect on resistance to lethal heat stress (35°C) of manipulation of AA levels using RNAi and AA supplementation. tdo-2(RNAi) reduces AA levels (this study, [37]) and increases Trp levels [37]. Restoration of AA in tdo-2(RNAi) did not restore stress sensitivity, nor did AA supplementation of wild-type worms increase stress sensitivity. kmo-1(RNAi) greatly increases AA levels (this study, [37]) and only marginally increases Trp levels [37]. Probability p of being the same as RNAi control calculated using the log rank test. Statistics: (A) RNAi control, N = 116, mean survival 15.5 h; tdo-2(RNAi), N = 111, mean survival 19.9 h, p<0.001. (B) RNAi control, N = 203, mean survival 11.6 h; kmo-1(RNAi), N = 178, mean survival 11.1 h, p = 0.0037; tdo-2(RNAi), N = 209, mean survival 12.2 h, p<0.001. (C) RNAi control, N = 220, mean survival 12.7 h; RNAi control+5 mM AA, N = 226, mean survival 12.6 h, p = 0.49; tdo-2(RNAi), N = 254, mean survival 13.3 h, p<0.001; tdo-2(RNAi)+5 mM AA, N = 249, mean survival = 249, p = 0.015. (D) Effect of mutations affecting kynurenine pathway on resistance to thermal injury (35°C). Only flu-2 mutants showed heat stress resistance. Statistics: wild type (N2), N = 100, mean survival 11.1 h; glo-1, N = 100, mean survival 12.3 h, p>0.05; flu-1, N = 100, mean survival 12.3 h, p>0.05; flu-2, N = 100, mean survival >16 h, p<0.001; nkat-1, N = 100, mean survival 12.9 h, p>0.05. (E) Effect of mutations affecting kynurenine pathway on resistance to osmotic stress (500 mM NaCl). glo-1 and flu-1 decrease AA levels, flu-1 mutation increases AA, while nkat-1 has little effect; however, all protect against lethal osmotic stress. Thus, there is no correspondence between AA level and resistance. Statistics: wild type, N = 300; glo-1, N = 250, p<0.01; flu-1, N = 150, p<0.01; flu-2, N = 150, p<0.05; nkat-1, N = 150, p<0.05. Probability p of being the same as wild type (two-tailed t test). * p<0.0 [file pbio.1001613.s017.pdf]

**A**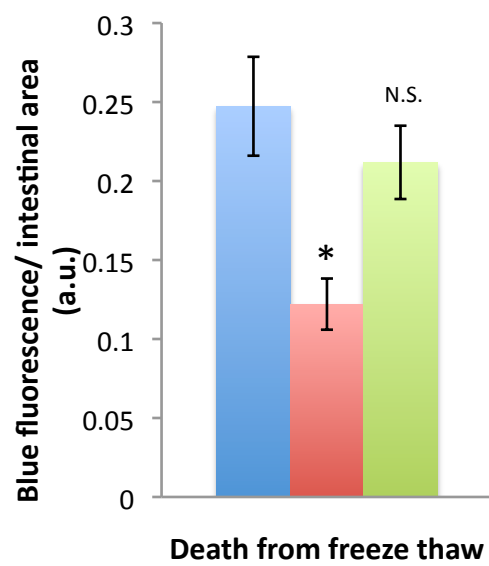**B**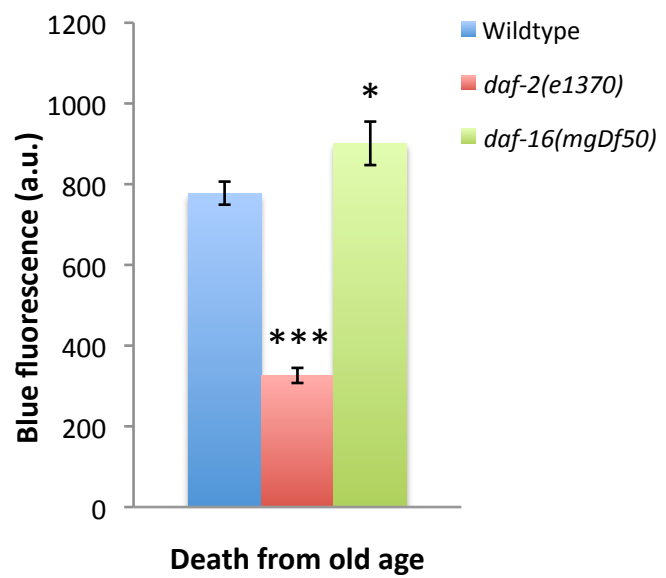

Supplement: Figure S18 — Effect of insulin/IGF-1 signaling on DF. (A) Death in young adults from lethal stress (freeze-thaw). Three biological replicates of 60 1-d-old adults each (measured in microtitre plates using plate reader). Fluorescence normalized to mean cross-sectional area of intestine. (B) Death in old adults from aging. Sample sizes: N2, 47; daf-2(e1370), 19; daf-16(mgDf50), 20 (measured in situ on NGM plates using time lapse photography). (PDF) [file pbio.1001613.s018.pdf]
