# Supplementary material for: Anthranilate Fluorescence Marks a Calcium-Propagated Necrotic Wave That Promotes Organismal Death in C. elegans
Source: PLoS Biol. 2013 Jul 23;11(7):e1001613. doi: 10.1371/journal.pbio.1001613 (PMC3720247; doi:10.1371/journal.pbio.1001613)
Supplement: Table S1 — Multiple types of fluorescence increase with death in C. elegans (cf., Figure S5). Table shows that both green and red fluorescence increase significantly with age, but blue fluorescence does not. Table also shows a significant increase in all types of fluorescence at death, with peak DF significantly higher than at any time seen during life. (DOCX) [file pbio.1001613.s019.docx]

**Table S1. Multiple types of fluorescence increase with death in *C. elegans*** (c.f. Figure S5).

| **Student’s *t* test *p* value** | **Blue** | **Green** | **Red** |
| --- | --- | --- | --- |
| Is the pre-death slope significantly greater than 0? | 0.0768 | 2.86e-14 | 2.35e-27 |
| Do the pre-death and post-death slopes differ significantly? | 5.93e-12 | 5.94e-13 | 1.56e-06 |
| Is the maximum pre-death fluorescence intensity significantly different from the peak death fluorescence intensity? | 1.02e-13 | 3.36e-19 | 1.82e-16 |

Table shows that both green and red fluorescence increase significantly with age, but blue fluorescence does not. Table also shows a significant increase in all types of fluorescence at death, with peak death fluorescence significantly higher than at any time seen during life.
