# Supplementary material for: Anthranilate Fluorescence Marks a Calcium-Propagated Necrotic Wave That Promotes Organismal Death in C. elegans
Source: PLoS Biol. 2013 Jul 23;11(7):e1001613. doi: 10.1371/journal.pbio.1001613 (PMC3720247; doi:10.1371/journal.pbio.1001613)
Supplement: Table S2 — NMR spectroscopic data for angl#1. (DOCX) [file pbio.1001613.s020.docx]

**Table S2. NMR spectroscopic data for angl#1**

| Position | angl#1 δ ^13^C [ppm] | angl#1 δ ^1^H [ppm] | angl#2^1^ δ ^1^H [ppm] | angl#1 ^1^H-^1^H-coupling  constants [Hz] | angl#1 HMBC correlations |
| --- | --- | --- | --- | --- | --- |
| 1 | 95.2 | 5.70 | 5.77 | J_1,2’’_ = 7.8 | C-2, C-3, C-4,  C-5, C-1’-COO |
| 2 | 73.8 | 3.50 | 3.67 |  | C-1, C-3 |
| 3 | 77.8 | 3.47 | 4.11  (J_H,P_ = 8Hz) |  | C-2, C-4 |
| 4 | 70.8 | 3.40 | 3.59 |  | C-5 |
| 5 | 78.5 | 3.44 |  |  | C-1, C-4 |
| 6a | 62.1 | 3.86 |  | J_6a,6b_ = 12.1,  J_5,6a_ = 5.2 | C-4, C-5 |
| 6b |  | 3.70 |  | J_5,6b_ = 2.1 | C-4, C-5 |
| 1’-COO | 167.8 |  |  |  |  |
| 1′ | 109.8 |  |  |  |  |
| 2′ | 153.1 |  |  |  |  |
| 3′ | 117.5 | 6.75 |  | J_3,4_ = 8.5, J_3,5_ = 1.1 | C-1’, C-5’,  C-1’-COO |
| 4′ | 135.5 | 7.26 |  | J_4,5_ = 7.1, J_4,6_ = 1.7 | C-2’, C-6’ |
| 5′ | 116.1 | 6.57 |  | J_5,6_ = 8.2 | C-1’, C-3’ |
| 6′ | 132.1 | 7.89 |  |  | C-2’, C-4’,  C-1’-COO |

^1^Characteristic ^1^H NMR signals of angl#2. ^1^H (600 MHz), ^13^C (151 MHz), and HMBC NMR spectroscopic data for angl #1 in methanol-*d*_4_. Chemical shifts were referenced to (CD_2_HOD) = 3.31 ppm and (CD_2_HOD) = 49.05 ppm.
