# Supplementary material for: Anthranilate Fluorescence Marks a Calcium-Propagated Necrotic Wave That Promotes Organismal Death in C. elegans
Source: PLoS Biol. 2013 Jul 23;11(7):e1001613. doi: 10.1371/journal.pbio.1001613 (PMC3720247; doi:10.1371/journal.pbio.1001613)
Supplement: Table S3 — NMR spectroscopic data for iglu#1. (DOCX) [file pbio.1001613.s021.docx]

**Table S3. Spectroscopic data for iglu #1**

| Position | iglu#1 δ ^13^C [ppm] | iglu#1 δ ^1^H [ppm] | iglu#2^1^ δ ^1^H [ppm] | iglu#1 ^1^H-^1^H-coupling  constants [Hz] | iglu#1 HMBC correlations |
| --- | --- | --- | --- | --- | --- |
| 1 | 86.5 | 5.46 | 5.55 | J_1,2_ = 9.0 | C-2, C-3, C-5,  C-2’, C-7a’ |
| 2 | 73.4 | 3.94 | 4.12 | J_2,3_ = 9.0, | C-1, C-3 |
| 3 | 78.9 | 3.60 | 4.24 (J_H,P_ = 8Hz) | J_3,4_ = 9.0 | C-2, C-4 |
| 4 | 71.2 | 3.50 | 3.68 | J_4,5_ = 9.0 | C-3, C-5, C-6 |
| 5 | 80.4 | 3.58 |  | J_5,6a_ = 5.8 | C-1, C-3, C-6 |
| 6a | 62.5 | 3.70 |  | J_6a,6b_ = 12.1 | C-4, C-5 |
| 6b |  | 3.88 |  | J_5,6b_ = 2.2 | C-4, C-5 |
| 2′ | 126.2 | 7.40 |  | J_2’,3’_ = 3.3 | C-1 (weak), C-3', C-3a’, C-7’ (weak), C-7a’ |
| 3′ | 103.2 | 6.49 |  |  | C-2’, C-3a’, C-4’ (weak),  C-7a’ (weak) |
| 3a′ | 130.3 |  |  |  |  |
| 4′ | 121.3 | 7.52 |  | J_4’,5’_ = 8.0, | C-3’, C-6’, C-7a’ |
| 5′ | 120.7 | 7.05 |  | J_5’,6’_ = 7.4, J_3,5_ = 1.1, | C-3a’, C-7’ |
| 6′ | 122.4 | 7.15 |  | J_6’,7’_ = 8.0, J_4’,6’_ = 1.0 | C-4’, C-7a’ |
| 7′ | 111.2 | 7.54 |  |  | C-3a’, C-5’ |
| 7a′ | 137.8 |  |  |  |  |

^1^Characteristic ^1^H NMR signals of iglu#2. ^1^H (600 MHz), ^13^C (151 MHz), and HMBC NMR spectroscopic data for iglu #1 in methanol-*d*_4_. Chemical shifts were referenced to (CD_2_HOD) = 3.31 ppm and (CD_2_HOD) = 49.05 ppm.
