# Supplementary material for: Anthranilate Fluorescence Marks a Calcium-Propagated Necrotic Wave That Promotes Organismal Death in C. elegans
Source: PLoS Biol. 2013 Jul 23;11(7):e1001613. doi: 10.1371/journal.pbio.1001613 (PMC3720247; doi:10.1371/journal.pbio.1001613)
Supplement: Table S4 — High-resolution MS data angl#1, angl#2, iglu#1, and iglu#2. (DOCX) [file pbio.1001613.s022.docx]

**Table S4. High-resolution MS data angl#1, angl#2, iglu#1, and iglu#2**

| **Compound** | **Ion** | **Ion formula** | **Calculated m/z** | **Observed m/z** |
| --- | --- | --- | --- | --- |
| angl#1 | [M-H]^-^ | C_13_H_16_NO_7_^-^ | 298.0932 | 298.0910 |
| angl#2 | [M-H]^-^ | C_13_H_17_NO_10_P^-^ | 378.0596 | 378.0600 |
| iglu#1 | [M-H]^-^ | C_14_H_16_NO_5_^-^ | 278.1034 | 278.1071 |
| iglu#2 | [M-H]^-^ | C_14_H_17_NO_8_P^-^ | 358.0697 | 358.0701 |

High-resolution MS data for previously unreported *C. elegans* metabolites angl#1, angl#2, iglu#1, and iglu#2, acquired using negative-ion electrospray ionization (ESI^-^).
